# Towards eliminating friction and wear in plain bearings operating without lubrication

**DOI:** 10.1038/s41598-023-44702-6

**Published:** 2023-10-13

**Authors:** Evgeny V. Kharanzhevskiy, Aleksey G. Ipatov, Aleksey V. Makarov, Faat Z. Gil’mutdinov

**Affiliations:** 1 Udmurt State University, Universitetskaya St., 1, Izhevsk, Russia; 2 Udmurt State Agricultural University, Studencheskaya St., 11, Izhevsk, Russia; 3 M.N. Mikheev Institute of Metal Physics of Ural Branch of Russian Academy of Sciences, S. Kovalevskaya St., 18, Yekaterinburg, Russia; 4 Institute of Engineering Science of Ural Branch of the Russian Academy of Sciences, Komsomolskaya St., 34, Yekaterinburg, Russia; 5 Ural Federal University, Mira St., 19, Yekaterinburg, Russia; 6 Udmurt Federal Research Center of Ural Branch of the Russian Academy of Sciences, T. Baramzinoy Str., 34, Izhevsk, Russia

**Keywords:** Mechanical engineering, Materials science

## Abstract

Plain bearings, renowned for their versatility and simplicity, are extensively utilized in engineering design across various industries involving moving parts. Lubrication is vital to the functioning of these bearings, yet their usage is inhibited under dynamic load conditions, or at elevated or reduced temperatures due to this dependency on lubrication. This study introduces an innovative method to significantly mitigate friction and wear in plain bearings operating without lubrication. The plain bearings were constructed from steel–bronze pairs, where the steel shafts were alloyed with bismuth oxide via short-pulse laser treatment. MnO_2_ was utilized as a carrier to incorporate the bismuth oxide into the surface layers of the steel. Insights from transmission electron microscopy and X-ray photoelectron spectroscopy revealed a highly non-equilibrium state of matter, unattainable through conventional engineering methods. The tribological performance of the modified steel disks was assessed via a block-on-ring sliding test, demonstrating superior wear and friction performance without lubrication, as well as an ultra-low coefficient of friction. Remarkably, the modified friction pairs remained functional after 200 km of linear sliding at a load of 250 N (12.5 MPa) and a sliding speed of 9 m/s. To substantiate the technique’s viability, we tested the performance of an internal combustion engine turbocharger fitted with a modified steel shaft. The turbocharger’s performance validated the long-term effectiveness of the steel–bronze coupling operating without lubrication at 75,000 rpm. The simplicity and resilience of this technique for modifying steel–bronze pairs offer a ground-breaking and promising approach for a wide range of applications.

## Introduction

Macroscale friction and wear continue to be primary sources of energy dissipation in moving mechanical components, with an estimated one-third of fuel being expended to overcome friction in automobiles alone. Moreover, wear significantly curtails the lifespan of mechanical components. Consequently, the quest to eliminate friction and wear has been unrelenting, highlighting the indispensability of achieving superlubricity. Initial realization of macroscale superlubricity under ambient conditions at room temperature^1^ paves the way for its application in real-world contexts, notably reducing energy consumption and mitigating CO_2_ emissions. Additionally, a pioneering method utilizing a single grain sliding at a nanoscale depth of cut and 40 m/s—speeds that are four to seven orders of magnitude higher than those in nanoscratching and micro-sliding—was developed^2^. Calculations and suggestions regarding force, stress, depth of cut, and size of plastic deformation were articulated for this innovative approach, thereby unveiling new avenues for exploring the fundamental mechanisms of sliding, grinding, and polishing^3–5^.

This imperative to understand and minimize friction and wear extends to the realm of plain journal bearings, also known as sleeve bearings or bushings, which are utilized in machinery to mitigate macroscale friction and wear between rotating shafts and stationary support members. Manufactured from materials such as bronzes, aluminum alloys, cast irons, and babbitt alloys, these bearings operate in tandem with steel shafts^6^. The engineering design and material selection for machinery employing plain bearings are contingent upon a replaceable, low-friction surface capable of withstanding the relative motion and forces inherent in sliding mechanisms^7^. Employed prolifically within the automotive industry—particularly in connecting rods, crankshafts, and camshaft assemblies of car engines—and in aircraft control systems, landing gear, and various engine components, including fuel pump shafts, plain bearings are prized for their simplicity, reliability, and capability to operate under high temperatures and dynamic loads. Furthermore, their usage spans to electric motors and generators, marine applications, hydroelectric power plants, industrial machinery, and home appliances, demonstrating their pervasive importance in various mechanical applications^6,7^.

The principal drawback associated with plain bearings hinges on the imperative nature of lubrication in their operational regime^7^. Diverse types of lubrication, such as boundary^8–10^, hydrodynamic^11,12^, and solid lubrication^13–15^, are employed, each proffering distinct advantages and appropriateness for varied applications^7^. Hydrodynamic lubrication, prevalent in high-speed applications, is preferentially utilized wherein solid lubricants are deemed ineffective due to suboptimal heat dissipation, augmented coefficients of friction (COF), and wear losses at elevated sliding speeds. While hydrodynamic lubrication mitigates direct interfacing between the bearing and shaft, its efficacy is reliant on the lubricative properties of machine oil or greases within a circumscribed operating temperature spectrum^7^. For example, the protracted operation of an engine turbocharger can be sustained within a temperature window of 50 to 105 °C for the motor oil in a combustion engine. Exposing these turbochargers to temperatures either above or below this range incurs irreversible damage resultant from inadequate lubrication, culminating in the seizure of the surfaces in interaction.

In specific situations, such as those involving very high contact pressures, low sliding velocities, or both low and elevated temperatures, hydrodynamic forces may be inadequate to sustain a continuous lubricant film between sliding surfaces. As a result, direct contact between the asperities might occur. This is particularly likely during start-stop operations or under similar conditions, prompting a transition to a different friction regime known as boundary lubrication^7^. Boundary lubrication is notable for its high friction coefficient^8–10,12^. This type of sliding can instigate significant wear, potentially to the point of galling, which might lead to seizure of the surfaces due to substantial junction growth that transpires during sliding with insufficient lubrication^8^. In such instances, the surfaces in contact can be protected by distinctive tribological layers, which form as a consequence of friction-induced energy dissipation between the two contact surfaces. These tribological layers need to have certain key properties, including low shear strength, strong film-forming capabilities, and the ability to transfer the formed films for self-lubricating materials^16^.

The primary drawback of tribological layers is their dependency on high temperatures, which activate the necessary chemical interactions with oxygen and associated oxygen diffusion processes^17^. This condition necessitates some heat-induced damage to the sliding surfaces prior to the transition to low friction. For instance, coatings based on boron carbides exhibit a transition to low friction only under high normal loads and high sliding rates when temperatures exceed 160 °C. In this case, the low coefficient of friction (COF) is linked to the formation of B–O–H bonds, resulting from the interaction of boron carbide in the coating with oxygen and moisture in the ambient air^18,19^. In another example, during the wear tests of nickel-zirconia coatings, intense sliding friction aids the creation of a third body via mutual mass transport from both surfaces in contact. This body, as a mechanically alloyed layer (MAL), prompts the transition to ultra-low friction, which only occurs at temperatures above 200 °C. Within the MAL, incorporated metals in their unoxidized state reduce the COF because adhesive forces and oxidation abilities between sliding bodies are successfully suppressed by the chemical composition of the MAL^20^.

The considerable practical benefits of tribological films, which significantly decrease friction and wear, are undeniable^21–28^. Utilizing materials with long-term self-lubrication capabilities in plain bearings could rival hydrodynamic lubrication, offering a pioneering and promising strategy in engineering design, especially for high-speed applications. Bismuth is one of the best candidates for forming a tribofilm between bronze and steel in a plain bearing. It does not undergo chemical interaction or mutual dissolution at both low and high temperatures and does not form solid solutions or intermetallic compounds with iron and copper^29^. However, an unresolved engineering challenge has been the inability to create iron-bismuth or copper-bismuth alloys, as these metals are completely immiscible, even in their liquid state^29^. This paper addresses this issue. The primary objective is to determine the impact of bismuth oxide, dispersed as nanoparticles in steel shafts, on friction and wear under conditions of severe sliding friction without lubrication at high loads and speeds. To corroborate the viability of this method, we assessed the performance of an internal combustion engine turbocharger fitted with a modified steel shaft. This demonstration aimed to showcase the persistent effectiveness of the modified steel–bronze coupling when operated without lubrication.

## Materials and methods

Test samples were manufactured with varying concentrations of bismuth oxide, dispersed as nanoparticles in the surface layers. A distinctive methodology known as “high-energy, short-pulse laser melting”^18,19^, was employed to integrate bismuth oxide into the steel matrix as nanoparticles. A distinguishing characteristic of this technology is the ultra-high rates of thermal cycling, capable of generating highly non-equilibrium states of materials, such as amorphous or nanocrystalline phases^30,31^. For instance, this technology can stabilize profoundly supersaturated solid solutions in equimolar Cu-Fe alloys^32^. The technique also allows synthesizing a broad spectrum of ceramic coatings on steels. An exceptional feature of the technique is a high level of adhesion between the substrate and ceramic coatings^18,19^.

Steel disks (C—0.4 wt%), each 10 mm thick and 75 mm in diameter, had their cylindrical surfaces alloyed with bismuth oxide via the laser processing. Before this laser treatment, a 30 μm-thick layer of fine powder mixtures, containing bismuth oxide (Bi_2_O_3_) and manganese oxide (MnO_2_), was sprayed onto the steel disks. The use of manganese oxide powder served as a carrier to provide the dispersion of bismuth oxide nanoparticles into the steel matrix. For each powder composition—0; 5; 10; 15; 17; or 20 wt% of Bi_2_O_3_ mixed with MnO_2_—five identical disks were fabricated. The powders had average particle sizes of 5 µm for Bi_2_O_3_ and 8 µm for MnO_2_, with a maximum size of a particle of 15 µm for any powder mixture. Steel disks for wear tests were produced from grade 40 carbon steel (containing 0.4% C as per GOST 1050–88 standard), which had a hardness of 34 HRC. The cylindrical surfaces of these disks were ground to reach a roughness Ra parameter of 0.32 µm.

The experimental arrangement for laser processing utilizes a short-pulse ytterbium fiber laser (λ = 1.065 μm, τ = 40 ns). We used a commercially available short-pulse laser, the LDesigner F1, which has a maximum power of 50 W. The laser treatment of the powder mixture on the steel surface was executed in a single pass, under an argon atmosphere with 99.997% purity. The pulse energy was set at 1 mJ at a frequency of 20 kHz, delivering an instantaneous power of 25 kW. With a laser beam focus diameter of 30 μm, the resulting instantaneous power density was measured to be 3.5 × 10^13^ W/m^2^. Laser processing was conducted by scanning the laser beam across the cylindrical surface of a disk. The detailed methodology and parameters for the laser beam movement and laser processing can be found in reference^18^. After the laser treatment, the surface roughness of the disk’s coated surface was reduced through diamond lapping, utilizing a spherical natural diamond tool with a 3 mm radius, which was spring-loaded with a force of 130 N.

The topography and chemical composition of wear surfaces were analyzed using the FEI Inspect S50 scanning electron microscope (SEM) running at 20 kV, coupled with an energy-dispersive X-ray (EDS) detector. The fine structure was observed with a transmission electron microscope (EM-125). The TEM samples were created by electropolishing foils in a mix of glacial acetic acid and chromic anhydride. The sample post-laser treatment surface was kept from being polished using a stainless-steel plate, leading to TEM samples being formed directly from the surface layers of the coating. These samples had a thickness of roughly 50 nm. The SPECS spectrometer fitted with a Phoibos-150 energy analyzer (part of the Centre of collective usage UdmFRC UB RAS’s equipment) was used for X-ray photoelectron spectroscopy (XPS) to evaluate the surface layer composition after wear tests. The CasaXPS software was used to compute the proportion of chemical constituents. Contaminants adsorbed onto the sample surfaces were cleaned using a stream of argon ions at a current density of 30 μA/cm^2^ and an energy level of 4 keV, directed at a 45° angle to the surface of the target. Surface etching was performed using the same method, and the argon-ion flow’s etching rate was between 0.9 and 1.1 nm per minute. Under these etching conditions, the chemical composition of the samples remained constant.

Wear tests were conducted in accordance with the ASTM G77 standard using a block-on-ring tribometer, designed to replicate the geometry of conformal contact area of sliding surfaces. The counterface, constructed of bronze (grade BrAFNM 10–4-2, as per Russian state standard GOST 1628–78), was applied against the cylindrical surface of a laser-treated disk using a normal force P. The loaded surface area of the samples measured 20 mm^2^ (5 × 4 mm). The normal force was adjusted between 50 and 450 N, with a consistent sliding speed of 9 m/s. Table 1 outlines the compositions of the counterface material employed in the wear tests. The chosen alloy is frequently utilized in the fabrication of high-speed plain journal bearings, common in various machinery applications.

**Table 1:** Composition of the counterface material.

Alloy	Standard	Composition (wt%)
Bronze BrAFNM 10–4-2	GOST 1628–78	Al—10; Fe—4; Ni—2; Mn—1; Cu—basis

All wear tests were implemented without lubrication, with a relative humidity maintained at 60 ± 5%. The absence of lubrication simulates the harsh conditions of significant oil deprivation, which is frequently encountered during machinery emergencies. The coefficient of friction (COF) was determined through the use of a force-measuring platform in which the counterface was mounted. The temperature during wear tests was measured by a type K thermocouple, installed on the side of the counterface, at a distance of 1 mm from the sliding surface. For each coating composition, a set of five identical wear tests were conducted to track the COF’s evolution and compute the mean square error. The specific wear rate ’*k*’ was calculated using the change in mass Δ*m* of the counterface due to wear, as represented in Eq. (1)^7^.

1 $$k = \frac{\Delta m }{{\rho LP}}.$$

Here, ‘*ρ*’ signifies the density of the sample, ’*L*’ indicates the distance traversed, and ‘*P*’ represents the normal load applied to the contact^7^. By quantifying the COF and wear rate ‘*k*’, we can investigate how varying the bismuth oxide content in the surface layers influences friction and wear during high-intensity sliding friction under high loads and sliding speeds.

## Results and discussion

As previously mentioned, the selection of bismuth as the primary element for a tribological layer is because of its complete immiscibility with iron and copper, even in their liquid state^29^. However, this means that alloying iron with bismuth to achieve a thermodynamically nonequilibrium state poses a significant challenge. Our tests have shown that short-pulse laser processing of bismuth oxide on the surface of steel samples results in a complete separation of bismuth and iron. The bismuth oxide powder is fully reduced by iron during the laser treatment. Given that bismuth in its metallic state has a melting point of 271.5 °C^33^, it segregates to the surface of the steel disk during the laser remelting process, creating a distinct metallic phase on the surface (see Supplementary Information Fig. S1).

**Figure 1:**
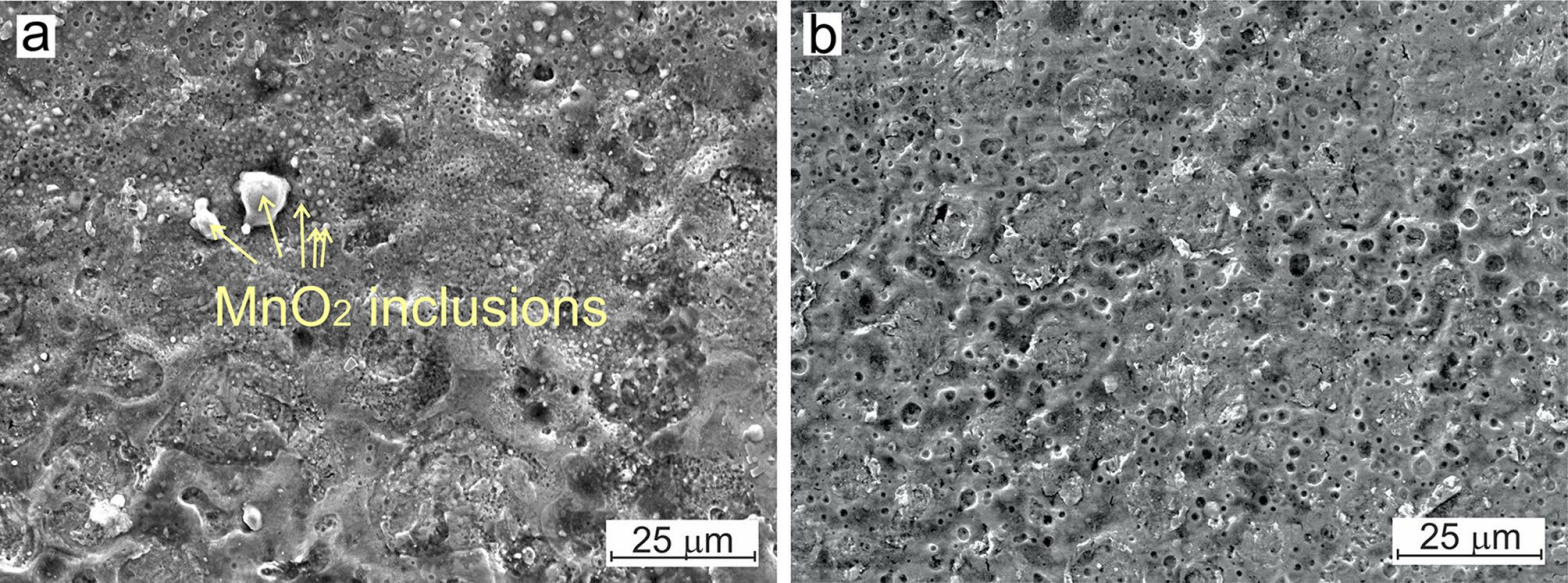
SEM-views of a steel disk surface after (**a**) laser short-pulse processing for a MnO_2_ – 20% Bi_2_O_3_ powder composition, and (**b**) consequent diamond lapping.

At temperatures above 450 °C, bismuth oxide reacts with some other oxides^33^, which could facilitate the dispersion of bismuth in the surface layers of steel. To solve this problem, we used manganese oxide powder, which we mixed with bismuth oxide in varying ratios. Results of the SEM investigation of a steel disk surface after laser processing with a MnO_2_—20% Bi_2_O_3_ powder composition and subsequent diamond lapping, are shown in Fig. 1. Numerous spherical-shaped inclusions, as indicated by arrows in Fig. 1a, were detected on the surface post-laser processing. According to the EDS analysis, these inclusions consist of manganese dioxide (MnO_2_), as depicted in Supplementary Information Fig. S2. Manganese dioxide has a low melting point of 535 °C and thus solidifies after the steel surface, forming spherical inclusions on the surface. Following diamond lapping of the surface as a post-laser processing (shown in Fig. 1b) removes all MnO_2_ inclusions from the surface, leaving the metal matrix solely with dissolved bismuth-containing oxides. EDS analysis indicate that after diamond finishing, in addition to Fe, the metal matrix contains elements such as Mn—1.66 at%, Bi—0.98 at%, and O—4.83 at%. In these quantities elements could form a complex manganese bismuth oxide, with a composition corresponding to BiMn_2_O_5_.

Diamond lapping removes MnO_2_ particles from the surface, leaving behind numerous spherical-shaped pores, as shown in Fig. 1b. When sliding with lubrication, these pores can retain the lubricant, positively impacting both friction and wear. This is akin to the tribological behaviors observed in laser-textured surfaces^34^.

**Figure 2:**
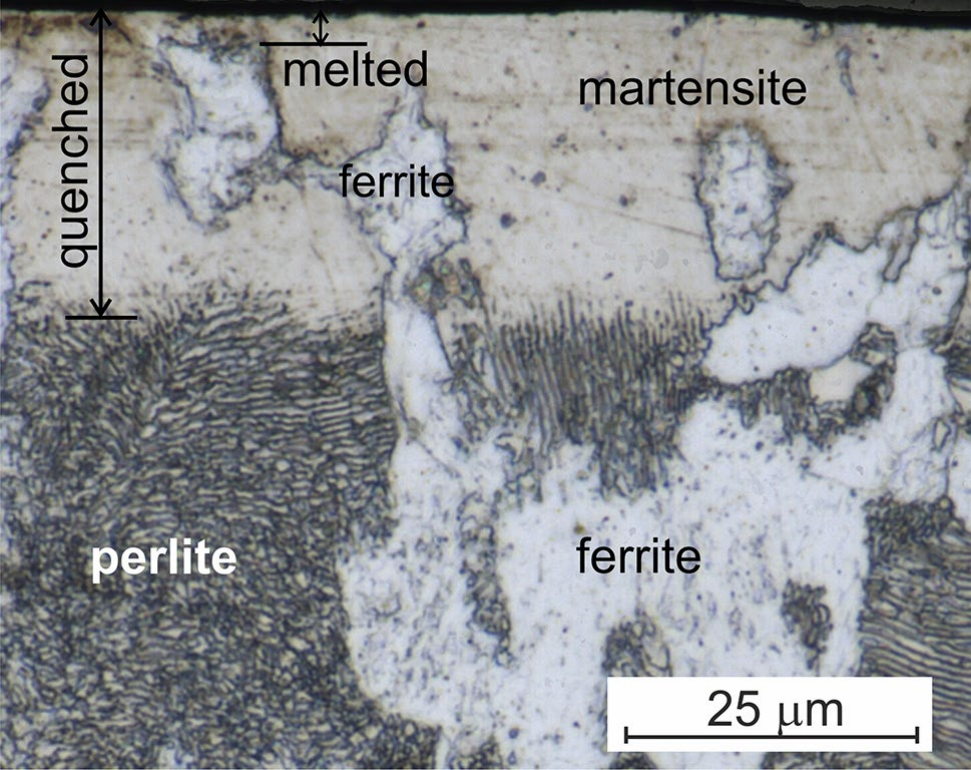
Cross section of a disk after laser treatment and diamond lapping for a MnO_2_—20% Bi_2_O_3_ powder composition.

The cross-section of a disk, after laser treatment and diamond lapping (as depicted in Fig. 2), exhibits a heat-affected area. This area consists of a solid-phase transformations zone and a melted zone. The microstructure of the steel disk in the state of delivery comprised ferrite and perlite grains. High-speed thermal heating and cooling, under the action of high-energy short laser pulses, result in the formation of martensite in place of the former perlite grains. However, the ferrite grains remain unchanged due to insufficient time for carbon solid-state diffusion during the short laser pulses. The brief duration of the laser pulse leads to a shallow depth of the melt pool, ranging from 3 to 5 mm, as depicted in Fig. 2. EDS analysis (see Supplementary Information Fig. S3) confirms that bismuth, manganese, and oxygen are detected only within a 3–5 µm thickness from the surface, coinciding with the melt layer’s thickness.

**Figure 3:**
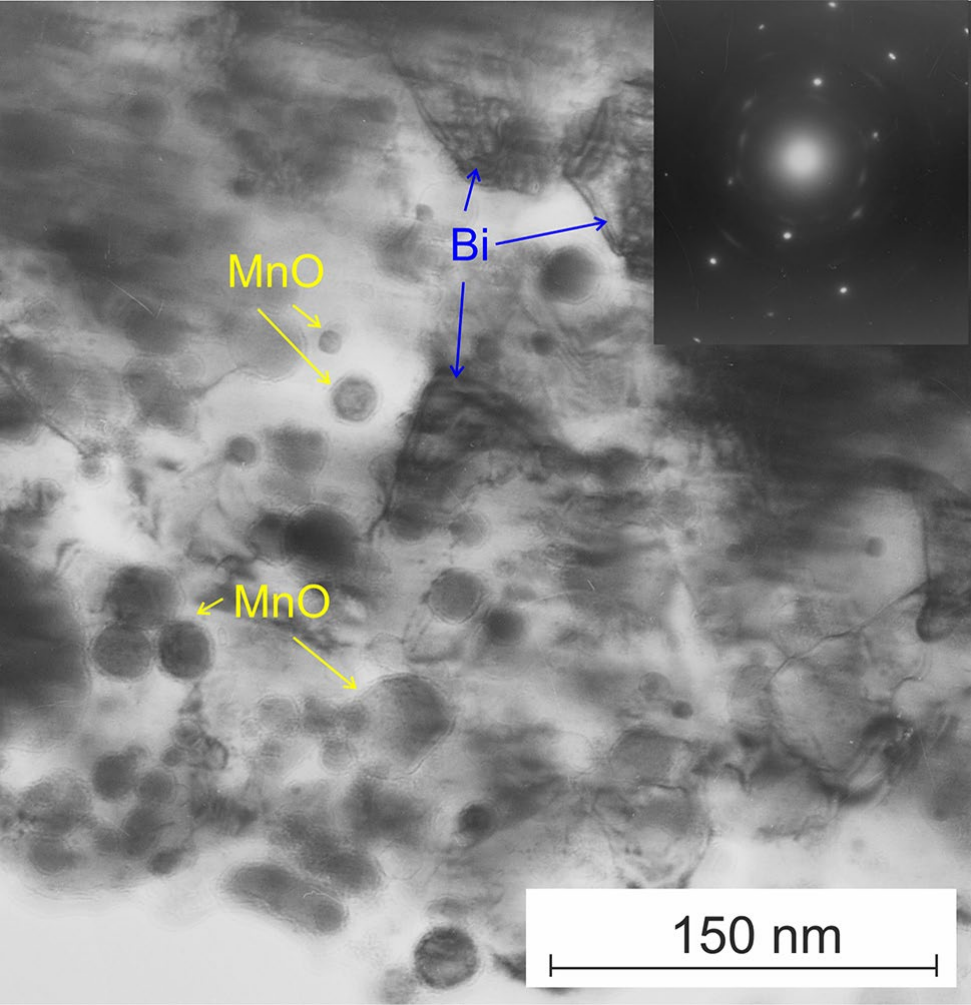
TEM-image and electron diffraction pattern (inset) of a sample subjected to laser processing with a MnO_2_—20% Bi_2_O_3_ powder mix.

Figure 3 presents the results from transmission electron microscopy applied to a sample that underwent laser processing with a MnO_2_—20% Bi_2_O_3_ powder mixture, followed by diamond lapping. The complex structure revealed is notably in a nonequilibrium state, showcasing a significantly distorted iron-based metal matrix punctuated by numerous nanoscale inclusions. Electron diffraction patterns deduced from the samples indicate that these inclusions consist of reduced bismuth in the metallic state and partially reduced manganese oxide (MnO) particles, which are spherically shaped. As indicated in Fig. 3, these high-contrast inclusions, highlighted by arrows, vary in size between 2 and 75 nm, markedly smaller than the initial particles of bismuth oxide and manganese oxide powder. These precipitates presumably formed during the binodal decomposition of the supercooled iron-oxides melt, preceding its rapid solidification. The structures generated during the initial phases of binodal or spinodal melt decomposition are characteristic for the employed high-energy, short-pulse laser melting process^32^.

The extraordinarily rapid cooling rate halts further melt decomposition, entrenching a supersaturated solid solution of normally disparate components in a solid phase^32^. In this context, the initially homogeneous iron-oxides melt partially decomposes into spherical liquid precipitates, which are oxide-rich and encased by a supersaturated oxides-iron melt. The ensuing rapid cooling solidifies these elements. Electron diffraction patterns expose the coexistence of ferrous iron oxide (+ 2) alongside metallic bismuth and MnO, both reduced from Bi_2_O_3_ and MnO_2_ by iron in the area. Adjacent to the inclusions, the emergence of transitional phases can be observed, as denoted by arrows in Fig. 3, illustrating a shell-like morphology. The presence of shell structures, iron oxides, and metallic bismuth corroborates the vigorous chemical interaction of manganese and bismuth oxide with iron in the molten zone during the high-energy short-pulse laser treatment. Attaining this nonequilibrium state is only possible under exceedingly high solidification rates that outpace the speed of absolute stability^35^ of the solid–liquid interface under the utilized processing conditions. Such conditions enable a planar type of crystal growth and trigger numerous nonequilibrium effects, such as diffusionless solidification and solute trapping^35^.

### Wear tests results

**Figure 4:**
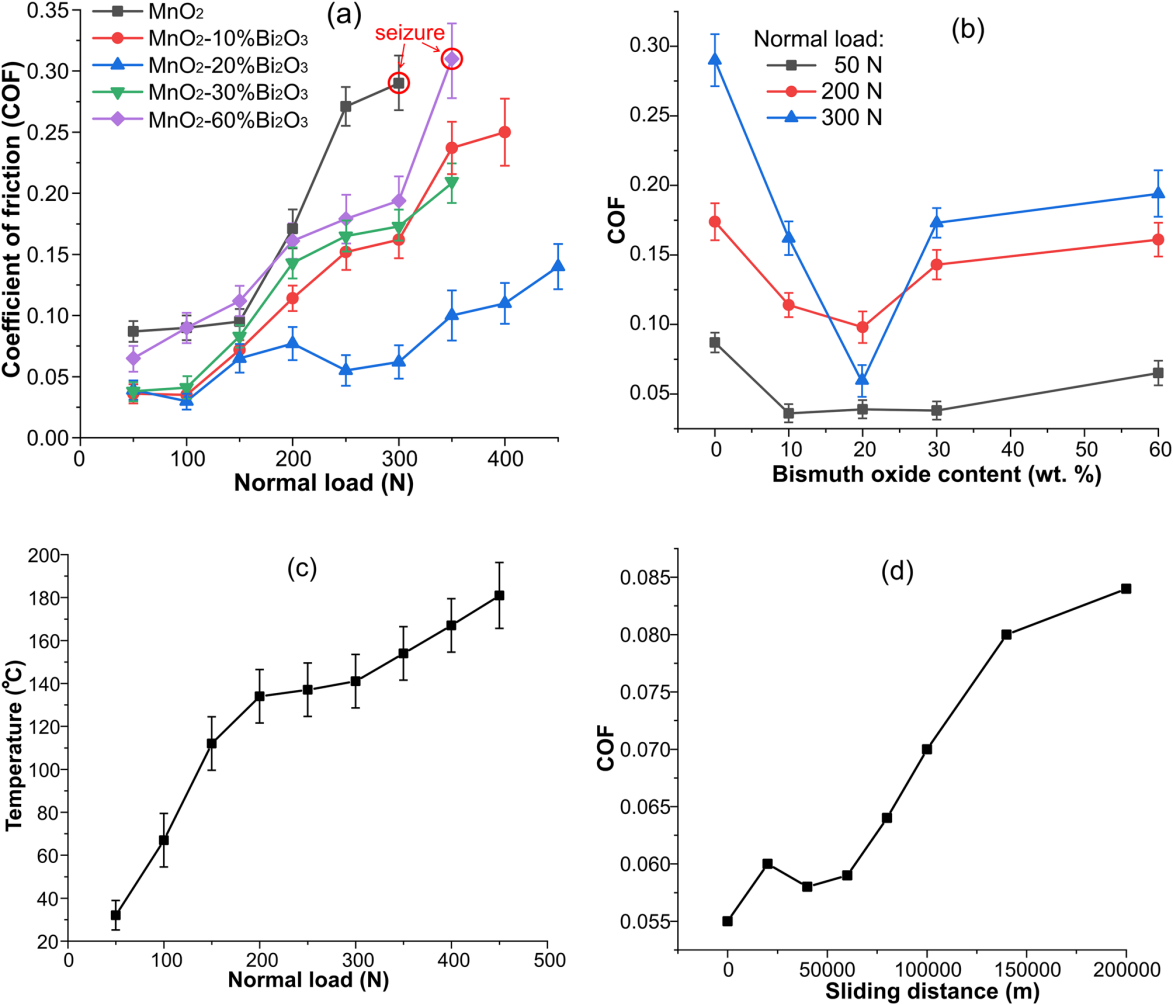
Wear tests results of oxide-alloyed steel disks sliding against bronze without lubrication: (**a**) evolution of COF depend on normal load for different oxide composition, (**b**) evolution of COF versus bismuth oxide content, (**c**) temperature evolution during wear test of disk alloyed with MnO_2_—20% Bi_2_O_3_ powder mix, (**d**) prolonged wear test of disk alloyed with MnO_2_—20% Bi_2_O_3_ powder mix at the normal load of 250 N.

The evolution of the COF during sliding wear, tested without lubrication across a broad range of normal loads applied to the samples, is depicted in Fig. 4. All samples, alloyed with various powder compositions, displayed a COF of less than 0.1 under normal loads up to 150 N (7.5 MPa). Even samples with solely dispersed manganese oxide demonstrated a positive impact on friction, exhibiting a COF of 0.09. However, at higher normal loads, the COF for these samples escalated sharply, reaching a value of 0.3. This corresponded to a transition to severe adhesive wear (galling) with further surface seizure noted in the case of MnO_2_-alloyed samples.

The introduction of bismuth oxide into the powder composition, coupled with subsequent short-pulse laser treatment of samples, profoundly alters their tribological performance. At lower normal loads, the registered COF values range from 0.036 to 0.045 for all samples, except for those alloyed solely with MnO_2_ or those with a MnO_2_—60% Bi_2_O_3_ powder mix (see Fig. 4b). As the load increases, samples generally exhibit an increased COF, with differing inclinations that depend on the content of the powder mix. The impact of bismuth oxide content in the powder mixes on the COF is illustrated in Fig. 4b. It is apparent that the optimal concentration of Bi_2_O_3_ is approximately 20 wt. %. For these samples, the COF remains ultra-low (below 0.1) across the wide range of normal loads (see Fig. 4a) even at high temperature (see Fig. 4c), as well as during prolonged operation without lubrication (see Fig. 4d).

A notable aspect of the tribological behavior of samples alloyed with complex oxide is their effective running-in capability. A low COF is registered from the very beginning of wear tests for all powder mix compositions. Such efficient running-in can be attributed to the presence of an initially formed tribological layer, potentially formed during diamond lapping after laser alloying. After running-in, this pre-formed tribolayer likely undergoes wear and disintegration under the stress of an increasing dynamic load. This results in a rise in the friction coefficient up to 0.18 at a load of 200 N for various alloying compositions. This load increase is accompanied by a temperature rise in the rubbed surfaces, as shown in Fig. 4c. The increase in temperature stimulates the formation of a new tribological layer, leading to further stabilization or even an abrupt drop in the COF, as observed in samples alloyed with a MnO_2_—20% Bi_2_O_3_ powder mix (see Fig. 4a).

Various theories exist to explain tribological layers formed in sliding contacts between two ductile materials. One theory focuses on Tribologically Transformed Structure (TTS), which results from plastic deformation and recrystallization on sliding surfaces, disregarding mass transport between two sliding bodies^21–28^. Another theory addresses the formation of Mechanically-Mixed Layers (MML) involving wear debris in interface layers, considering mass transport and chemical interaction with the surrounding atmosphere^22–24,36,37^. Typically, MMLs form at low temperatures, excluding direct contact between the two sliding surfaces^38,39^, acting as solid lubricants. MMLs have demonstrated the ability to substantially reduce friction and wear under dry conditions^40^.

**Figure 5:**
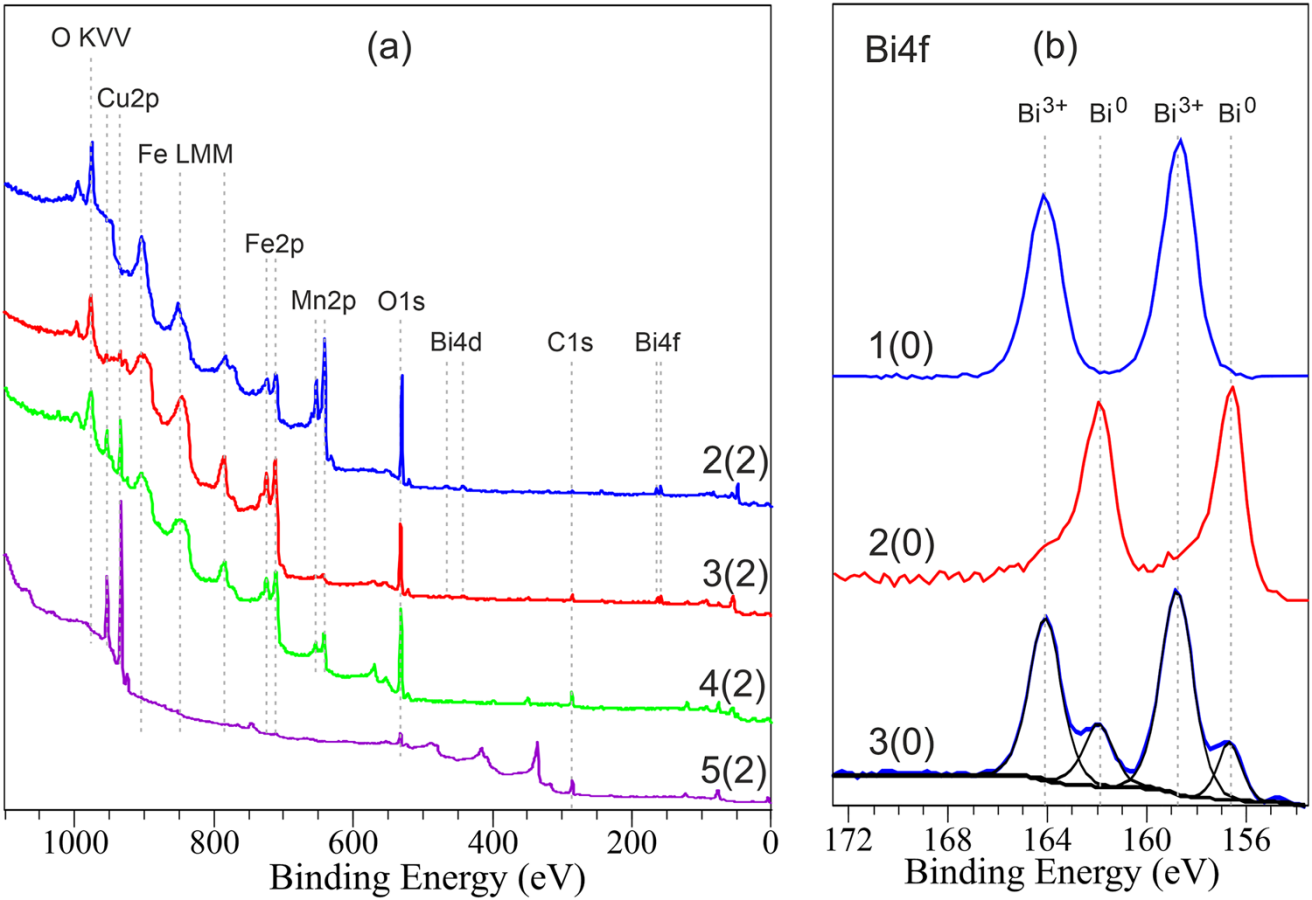
XPS spectra of the samples: Overall spectra (**a**) and spectra (**b**) in the region of bismuth (Bi4f). Numbers in brackets correspond to etching depths in nm by Ar-ions: 1—Spectrum of MnO_2_—20% Bi_2_O_3_-alloyed steel disk after laser alloying; 2—Spectrum of the same disk after diamond lapping; 3—Spectrum of the same disk after wear test at a load of 450 N; 4—Spectrum of the MnO_2_-alloyed steel disk after wear test at a load of 250 N; 5—Spectrum of the bronze counterface after the wear test in conjunction with the MnO_2_—20% Bi_2_O_3_-alloyed steel disk.

To characterize the tribological layers formed on contact surfaces, XPS analysis was conducted. The XPS spectra (Fig. 5) reveal the chemical state of materials on the surface of the sliding bodies. From the spectra and the assessment of the quantitative composition of the surface layers, it can be inferred that the surface of laser-alloyed steel disks consists of iron-based oxides, with dissolved manganese and bismuth cations. According to the XPS data (Fig. 5b, curve 1), the Bi4f spectrum of Bi-alloyed samples post-laser processing corresponds to the oxidation state of bismuth 3+ (the peaks of the spin doublet have values of *E*_*b*_ = 158.9 and 34.3 eV, according to the NIST database). After ion etching, the positions of the main maxima of the Bi4f spin doublet are maintained, but signs of metallic bismuth appear to the right of each of them. This is likely a result of partial reduction during Ar ^+^ -ion etching under conditions of ultrahigh vacuum (low partial pressure of oxygen).

Completely different XPS spectra are observed after the diamond lapping of Bi-alloyed samples (see Fig. 5b, curve 2). These spectra from surface correspond to photoemissions from bismuth in the metallic (Bi^0^) state. Therefore, it can be inferred that diamond lapping of samples with a spherical diamond tool results in the reduction of bismuth oxide in the thin surface layers (up to the depth of XPS analysis) to the metallic state. This reduction of bismuth oxide could occur due to a mechanical impact similar to the intensive plastic deformation experienced by thin surface layers.

After the wear test, the XPS spectra of Bi-alloyed samples taken from the surface prior to ion etching display the spin doublet from Bi^0^ with binding energies of 156.6 and 161.9 eV (± 0.2 eV). Additionally, the 4f. spin doublet from Bi^3+^ (158.8; 34.1 eV) is depicted by spectrum 3 in Fig. 5b. After 2 min of ion etching to a depth of about 2 nm, the Bi4f spectrum closely resembles that of pure metallic bismuth. Therefore, it can be inferred that the sliding friction of Bi-alloyed steel disks leads to the partial oxidation of bismuth on the rubbed surfaces. The resulting bismuth oxide readily reacts with atmospheric carbon dioxide to produce bismuth subcarbonate (BiO)_2_CO_3_^41^, which has a layered structure consisting of (BiO)n-cations surrounded by (CO_3_)-anions. This layered structure easily shears during friction, thus providing an ultra-low coefficient of friction (COF). The XPS spectrum of the bronze counterface, post-wear test in conjunction with the MnO_2_-20% Bi_2_O_3_-alloyed steel disk, reveals no signs of bismuth transfer from the alloyed steel disk (see Fig. 5a, curve 5). This allows us to conclude that there is no mass transport occurring between the two sliding bodies during the wear tests. The friction test results in an increase in the concentration of bismuth on the rubbed surface of the steel disk. Consequently, the tribological layers can be attributed to TTS.

**Figure 6:**
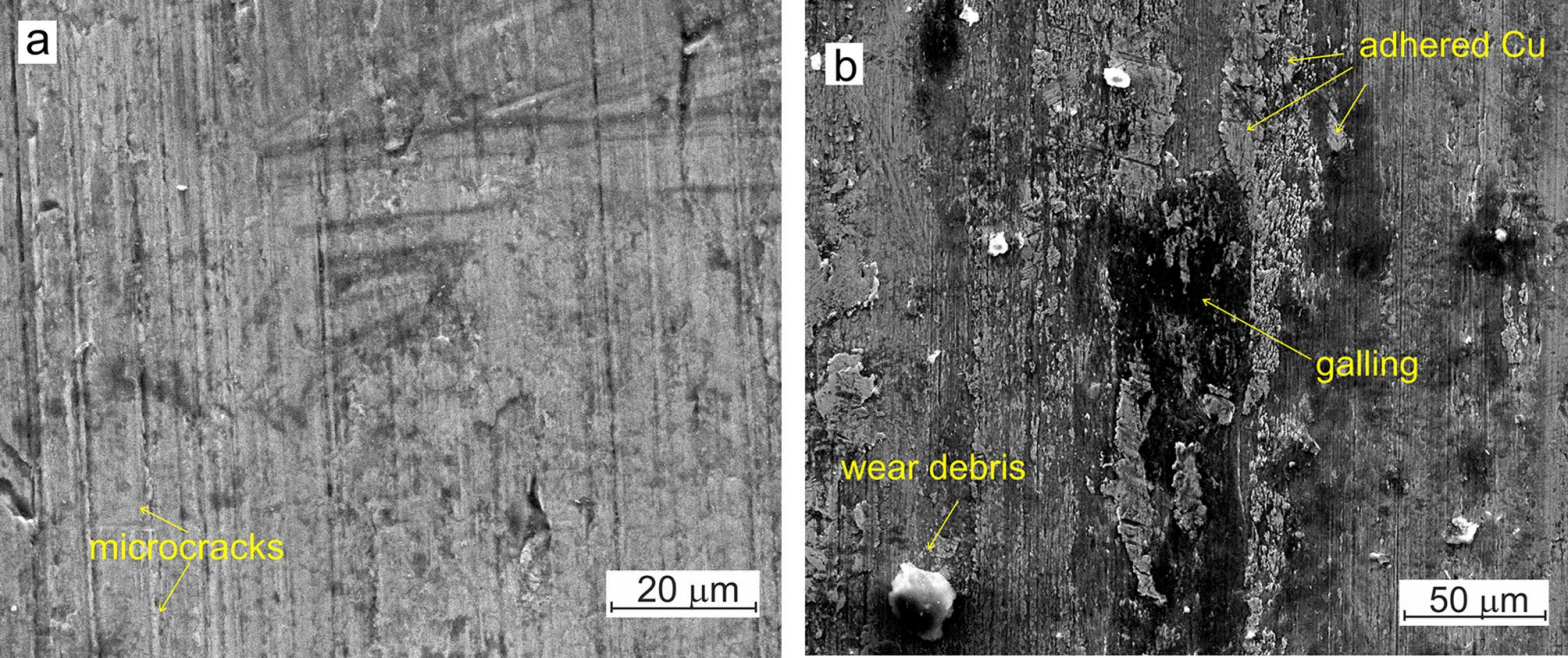
Surfaces of steel disks after wear tests conducted at a normal load of 250 N, sliding at 9 m/s, without lubrication: (**a**) MnO_2_—20% Bi_2_O_3_ alloyed steel disk after sliding for a distance of 200 km; (**b**) Steel disk alloyed solely with MnO_2_, after sliding for a distance of 1.2 km.

Insights from EDS analysis of surfaces after wear tests also reveal that laser alloying of steel with Bi prevents surfaces from mutual mass transport during sliding friction of steel–bronze pairs without lubrication, even after long-term tests (200 km), as shown in Fig. 6a. In contrast, MnO_2_-alloyed steel disks show copper adhered from the counterface, as seen in Fig. 6b. Adhesive forces cause severe damage to the MnO_2_-alloyed steel disk, indicating galling of the surfaces in contact even after sliding distance of 1.2 km. Severe sliding friction of this pair causes increased specific wear of the counterface, in relation to friction with Bi-alloyed steel disk, as shown in Table 2.

**Table 2:** Specific wear rate *k* (mm^3^/m N) of bronze counterfaces at the end of the wear tests calculated by Eq. (1).

Materials of disks	Load, sliding speed	Wear distance (m)	Specific wear rate *k* (mm^3^/m N) of a bronze counterface
Steel disk	150 N, 3 m/s*	6000	2.08 × 10^–4^
MnO_2_—alloyed disk	250 N, 9 m/s	18,000	1.31 × 10^–4^
MnO_2_—20% Bi_2_O_3_—alloyed disk	250 N, 9 m/s	200,000	0.50 × 10^–4^
MnO_2_—20% Bi_2_O_3_—alloyed disk	450 N, 9 m/s	18,000	0.74 × 10^–4^

*The load and the sliding speed for this test pair were reduced to withstand friction in absence of lubrication without seizure.

Fatigue failure of parts and kinematic conjunctions is one of the most critical issues in civil and mechanical engineering. Addressing this issue is essential for ensuring the long-term, reliable operation of machinery. In hydrodynamic lubrication, energy dissipation occurs through the internal friction of oil, and any excessive heat generated can be effectively removed by coolers. In contrast, when self-lubrication occurs by the formation of tribological layers on contacting surfaces, the incoming energy—which is equal to the work of the friction force—can cause fatigue failure of those surfaces. A long-term wear test, visualized in Fig. 4d, was performed at a normal load of 250 N (12.5 MPa). After a sliding distance of 200 km at a speed of 9 m/s, the surface of the steel disk alloyed with MnO_2_—20% Bi_2_O_3_ exhibits tiny microcracks. This demonstrates fatigue damage to the surface, as shown in Fig. 6a. Figure 6b shows the worn-out surface of a disk alloyed solely with MnO_2_. The damage to the surface of the steel disk, including galling and vast areas of adhered copper from the counterface, is also illustrated in Fig. 6b.

### Turbocharger performance tests

**Figure 7:**
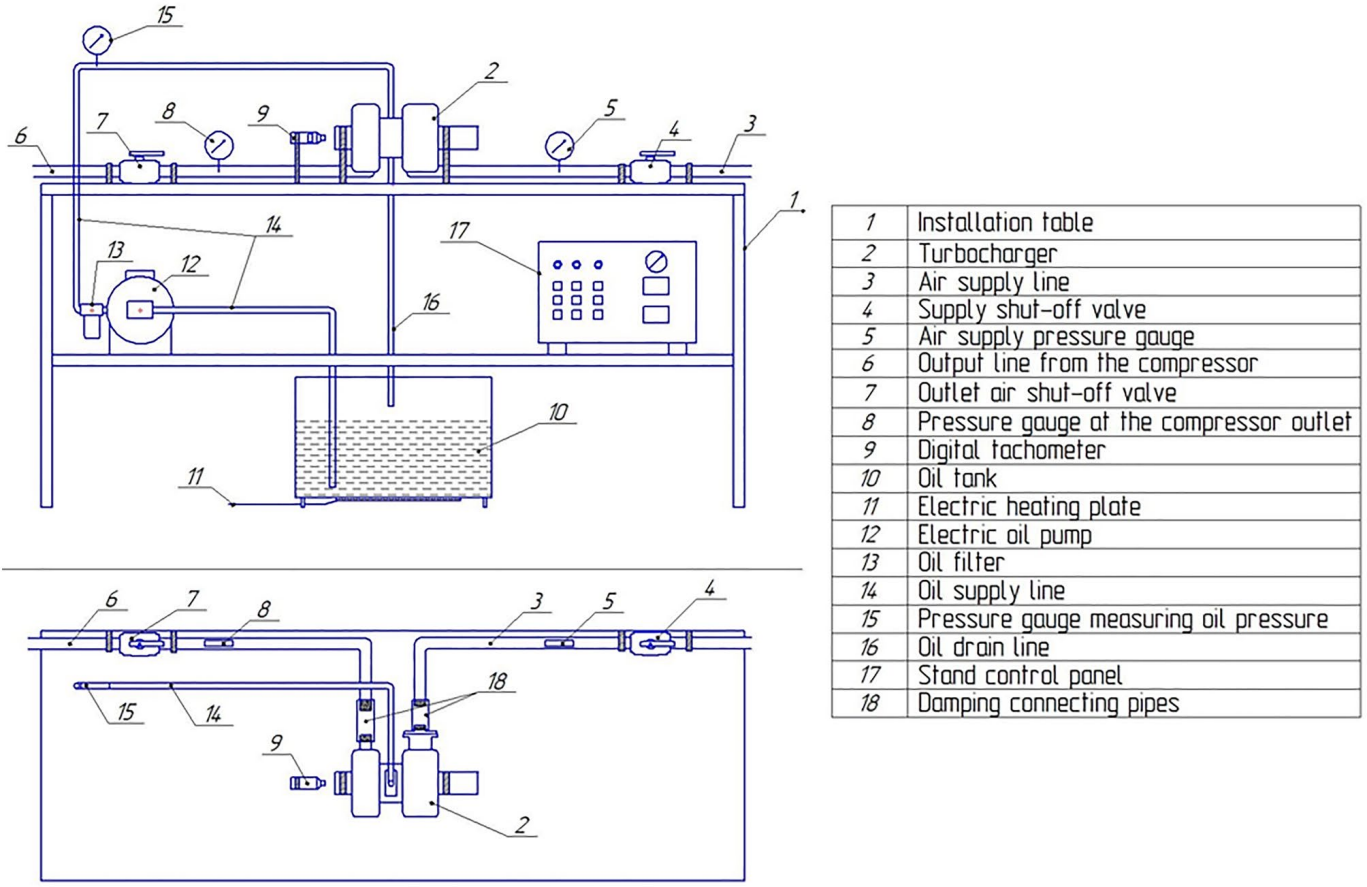
Schematic representation of the laboratory test stand, illustrating the setup and arrangement of key equipment and measurement devices utilized during the experimentation phase.

To investigate the influence of bismuth alloying on a steel shaft of the bearing assembly, a laboratory test stand was developed for turbocharger tests (Fig. 7). This laboratory setup can spin the turbocharger’s shaft up to 100,000 rpm under the influence of compressed air, supplied from the compressor reservoir at a pressure of 8–10 atmospheres. During testing, the lubrication conditions of the turbocharger’s bearing assembly can be fully simulated. The lubrication system is autonomous, featuring a heater (11) and an electric pump (12) for the delivery of lubricating material to the bearing assembly. The following are analyzed during testing: The air flow rate into the turbine’s impeller, using an anemometer (5) built into the air supply line (3); The compressor wheel’s rotational speed, using an electronic tachometer (9); The degree of pressure increase in the compressor’s channel was controlled using a pressure gauge (8). The primary turbocharger performance indicator is the turbocharger shaft’s stoppage time after interruption of compressed air supply according to standard GOST 53637–2009.

Testing of the Mitsubishi Turbo turbocharger model Iveco MFD 49135–05122 in a delivery state (without alloying of the plain bearing assembly) proceeds as follows: the test turbocharger (2, Fig. 7) is fixed on the installation table (1), and the oil supply and drain channels are connected. Compressed air supply lines (3) and the outlet line (6) are connected using damping connecting pipes (18). The lubricating oil heating is activated. When the temperature reaches 40 °C, the electric pump (12) for delivering the lubricating material to the turbocharger’s bearing assembly is switched on for 5 s. After this period of time the oil lubrication pump is turned off, and immediately with that the valve (4) is opened to accelerate the compressor wheel to 75,000 rpm. Once the required turbocharger shaft rotational speed is achieved, the valve (4) automatically closes. After turning off the compressed air, the turbocharger shaft’s stoppage time was measured using an electronic tachometer (9). The turbocharger shaft rotation without excessive oil pressure in the bearing assembly simulates boundary lubrication.

**Figure 8:**
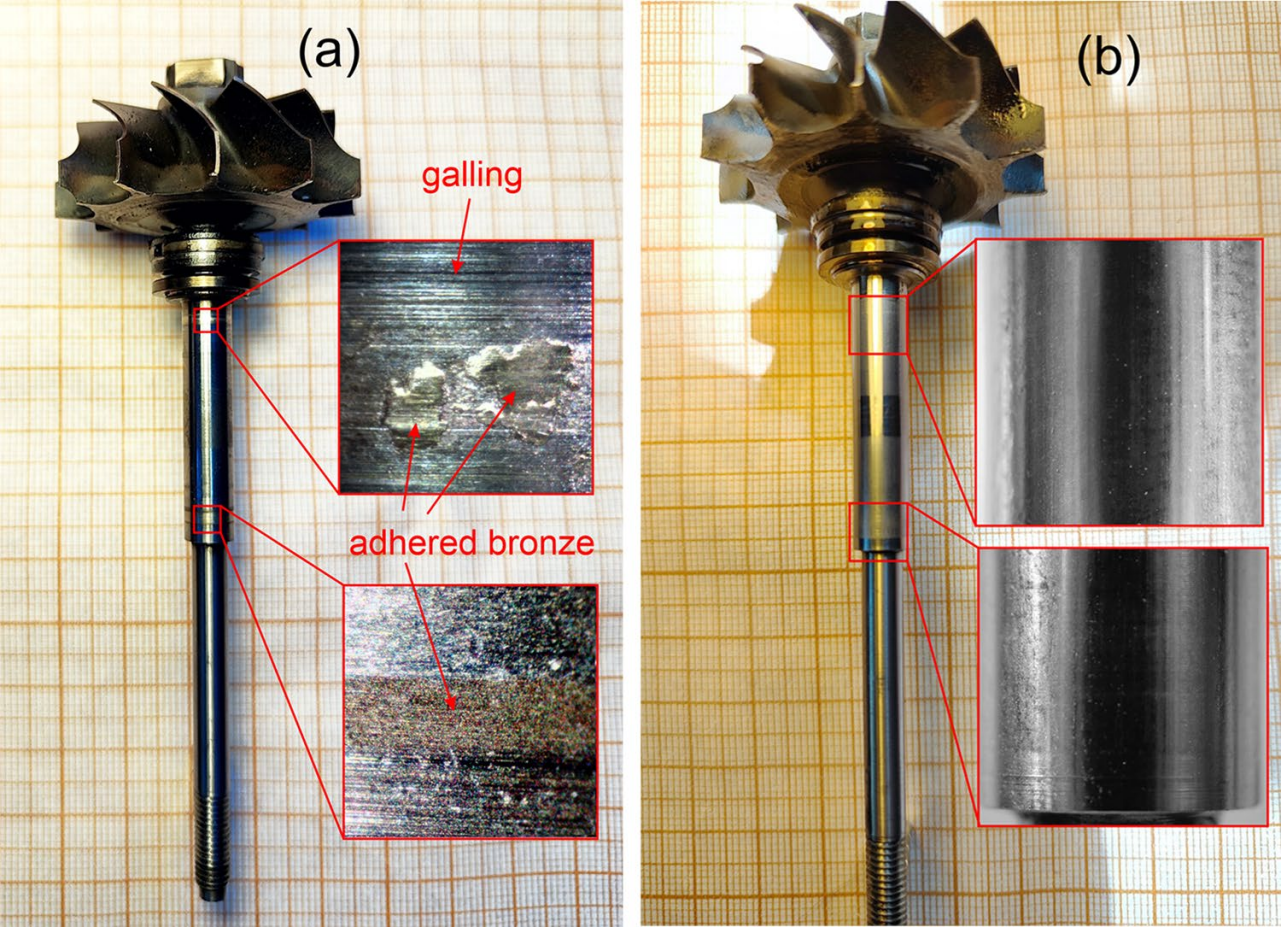
Steel shafts of turbochargers after friction tests: (**a**)—Steel shaft in its state of delivery after tests at 75,000 rpm in boundary lubrication for 5 cycles; (**b**)—Bismuth-alloyed steel shaft after tests at 75,000 rpm without lubrication for 500 start-stop cycles.

The tests of the turbocharger with unalloyed steel shaft were repeated 5 times, because during the fifth test an increase in the shaft vibration was registered. After this testing phase the turbocharger was disassembled for observation of caused damage. The steel shaft after tests is shown in Fig. 8a. Insets show severe damage of steel shaft in the places of the contact points with the bronze bushings. Adhered bronze and sights of galling are clearly visible on a macroscale.

Friction tests of a turbocharger fitted with a modified steel shaft were performed in an enhanced mode to evaluate performance capabilities. Bismuth alloying and subsequent diamond lapping of the steel shaft were carried out at the contact points with the bronze bushings. The subsequent testing phase of the modified turbocharger was conducted under the following conditions:

- The rotation speed was set to 75,000 rpm;
- Each test cycle consisted of 3 phases: acceleration of the turbine shaft, steady motion at 75,000 rpm for 20 s, and runout;
- The tests were performed entirely without lubrication at all stages;
- The number of test cycles was set to 500. All 500 cycles of the test were completed over two days.

After the test, the modified turbocharger was disassembled for observation. The bismuth-alloyed steel shaft after 500 cycles of testing without lubrication is shown in Fig. 8b. Thorough observation (see insets in Fig. 8b) demonstrates the absolute absence of any damage to the shaft. Therefore, we can conclude that bismuth alloying eliminates adhesion and mass transport between sliding bodies, providing outstanding antifriction properties and long-term reliability when sliding at high speed (75,000 rpm) without lubrication.

**Figure 9:**
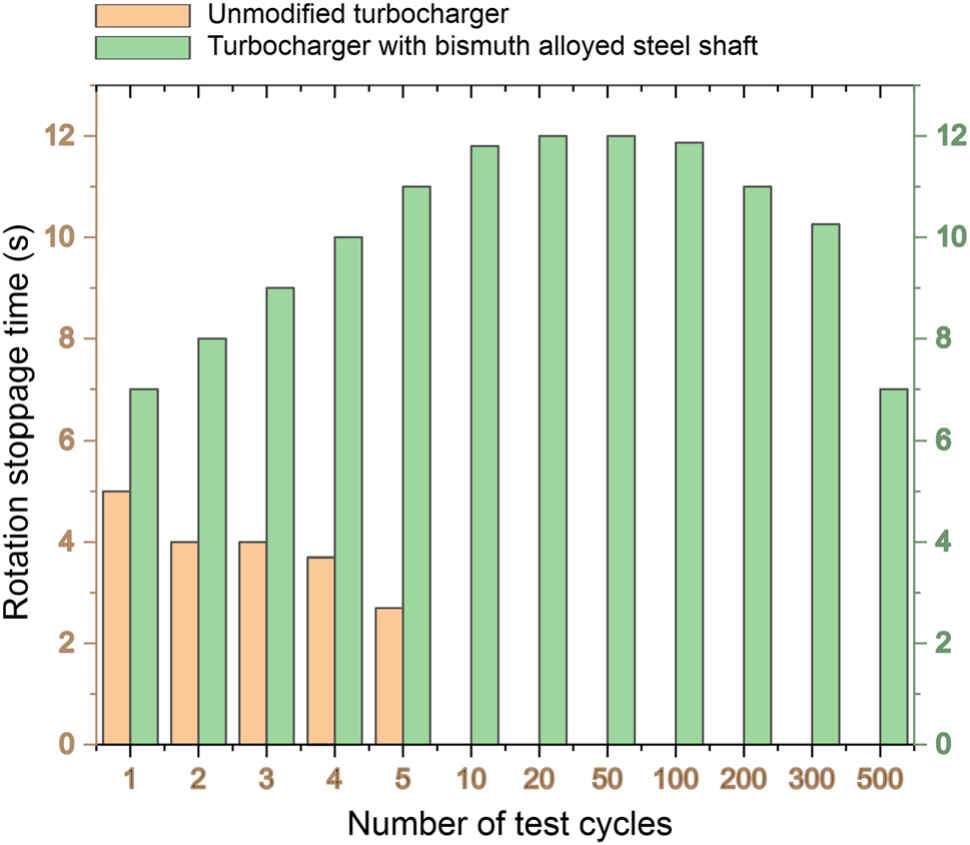
Rotation stoppage time of unmodified and modified turbochargers.

Results of turbochargers shafts stoppage time measurements for modified and unmodified turbochargers are shown in Fig. 9. Unmodified turbocharger endures only 5 cycles even in boundary lubrication. Contrariwise, the turbocharger with bismuth-alloyed steel shaft shows possibility working without lubrication for at least 500 cycles. After tests the modified turbocharger remained working. First 5 cycles of tests in Fig. 9 shows different trends for the modified and unmodified turbochargers. While the turbocharger in the state of delivery demonstrates an immediate decrease in the shaft stoppage time with number of cycles, the turbocharger fitted with a bismuth-alloyed steel shaft shows an increase in the stoppage time.

The increase in the rotation stoppage time of the alloyed shaft indicates excellent surface run-in ability. After 100 cycles of testing, the stoppage time began to decay gradually, accelerating after the 300th cycle. This decrease in the stoppage time coincided with a relatively minor increase in vibrations. Upon completion of the 500 cycles, an examination of the bronze bushings revealed an increase in the inner diameter by 0.081 mm due to wear loss. Bronze bushings, being easy to replace, are much more affordable compared to the turbine shaft. Furthermore, the need to replace bronze bushings is a minor concern for the tested turbine assembly operated in an emergency mode for two days without lubrication at 75,000 rpm. This exceptional emergency-resistant property also provides an outstanding opportunity to significantly extend the life cycle of machinery across various fields of mechanical engineering.

The procedures of laser alloying and subsequent diamond lapping are efficient and expedient. The complete alloying process of the turbocharger shaft, using bismuth, consumes a duration of slightly over 5 min. Through the implementation of advanced process optimization methods and the utilization of contemporary high-frequency, short-pulse lasers, the duration required for laser alloying can be significantly minimized. The finishing operation, diamond lapping, is even more time-efficient, necessitating slightly over 3 min. The introduced methodology is compatible with high-volume production applications in various mechanical engineering domains relevant to civilian sectors, including aerospace, automotive, and energy industries. Thus, this innovative technique for modifying steel–bronze pairs stands out for its cost-effectiveness, simplicity, and robustness, and it lends itself well to mass production. As such, it presents a revolutionary and promising pathway for a multitude of applications in mechanical engineering design. This method significantly mitigates the current limitations associated with lubrication-dependent bearings.

The technique for bismuth alloying presented can be applied for all types of lubrication. In the case of hydrodynamic lubrication, it can be useful in machinery operating in start-stop regimes, or in instances where the oil cannot maintain the necessary film thickness, such as at extremely low or high temperatures. For boundary lubrication, it can prevent damage caused by contact between the asperities of sliding bodies and significantly reduce the coefficient of friction. In the absence of lubrication, it can maintain an ultra-low coefficient of friction as low as 0.03, even at high speeds and loads. However, in this situation, the normal loads and contact pressures should be reduced to a level below the fatigue limit of shear stresses. This adjustment enables an infinite number of loading cycles or shaft rotations to be applied to a material without causing shear fatigue failure or significant wear loss.

## Conclusion

Despite being entirely immiscible with iron or copper, even in their liquid state, bismuth can be alloyed with steel through advanced short-pulse laser treatment, achieving a highly non-equilibrium state of matter. MnO_2_ has proven to be an effective carrier, facilitating the incorporation of bismuth oxide into the steel’s surface layers. In steel, bismuth manifests as nanoscale inclusions of metal and oxide particles.

Bismuth-alloyed steel shafts have demonstrated superior wear and friction resistance in the absence of lubrication, exhibiting an ultra-low coefficient of friction. An optimal powder composition for laser alloying includes MnO_2_—20% Bi_2_O_3_. This composition provides an ultra-low COF across a wide range of normal loads at high sliding speeds. The transition to ultra-low friction occurs through a two-fold mechanism: firstly, the surface bismuth, due to its immiscibility with copper, prevents adhesion between sliding bodies. Secondly, the bismuth oxide on the surface readily reacts with CO and CO_2_ in the surrounding air to produce subcarbonate (BiO)_2_CO_3_, which easily shears during friction, yielding an ultra-low coefficient of friction.

Contrary to solid lubricants, like those based on MoS_2_, graphite, and Teflon, bismuth-alloyed steel shafts maintain efficacy under conditions of high loads, linear velocities, and rotational speeds, as encountered in turbines. The long-term efficiency of the steel–bronze coupling, operating without lubrication at an impressive 75,000 rpm, was confirmed through the performance of an internal combustion engine turbocharger fitted with a modified steel shaft.

### Supplementary Information

Supplementary Information.

## Data Availability

All data generated or analyzed during this study are included in this published article and its supplementary information file.
