# Supplementary material for: Towards eliminating friction and wear in plain bearings operating without lubrication
Source: Sci Rep. 2023 Oct 13;13:17362. doi: 10.1038/s41598-023-44702-6 (PMC10576080; doi:10.1038/s41598-023-44702-6)
Supplement: Supplementary file 1 — Supplementary Information. [file 41598_2023_44702_MOESM1_ESM.docx]

**Supplementary Information and RAW DATA**


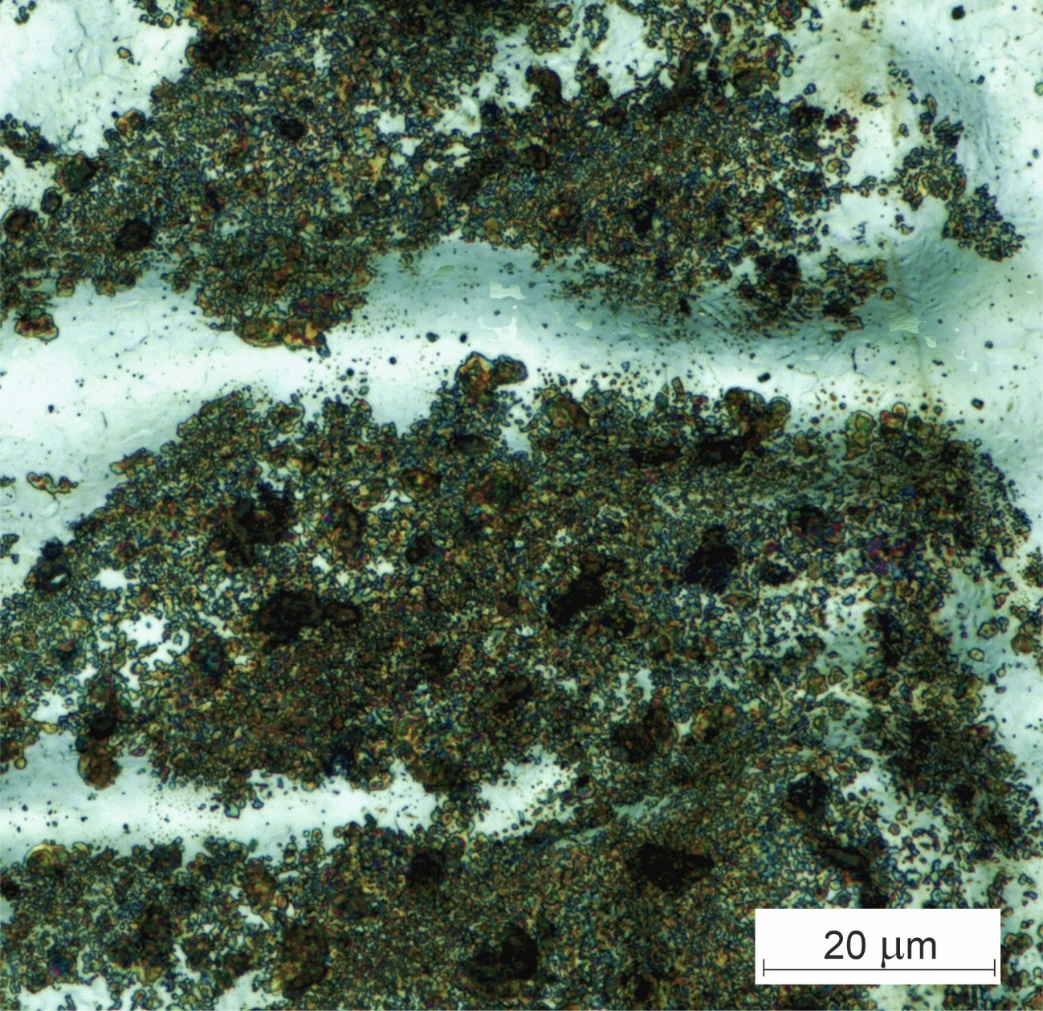


Fig. S1. Optical image of the surface of a steel disk after short-pulse laser alloying with Bi_2_O_3_ powder.


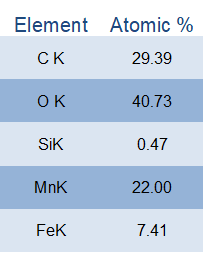

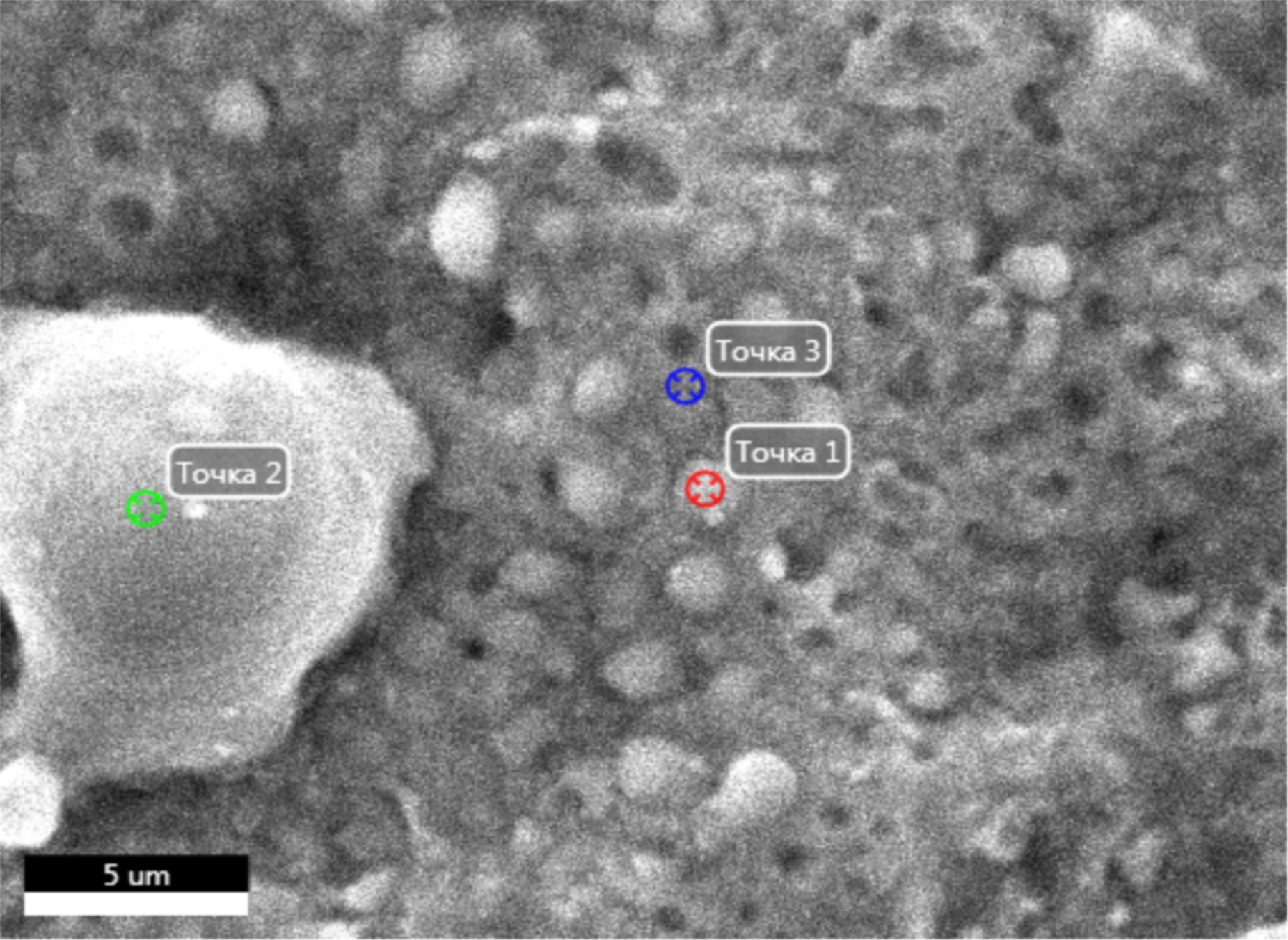

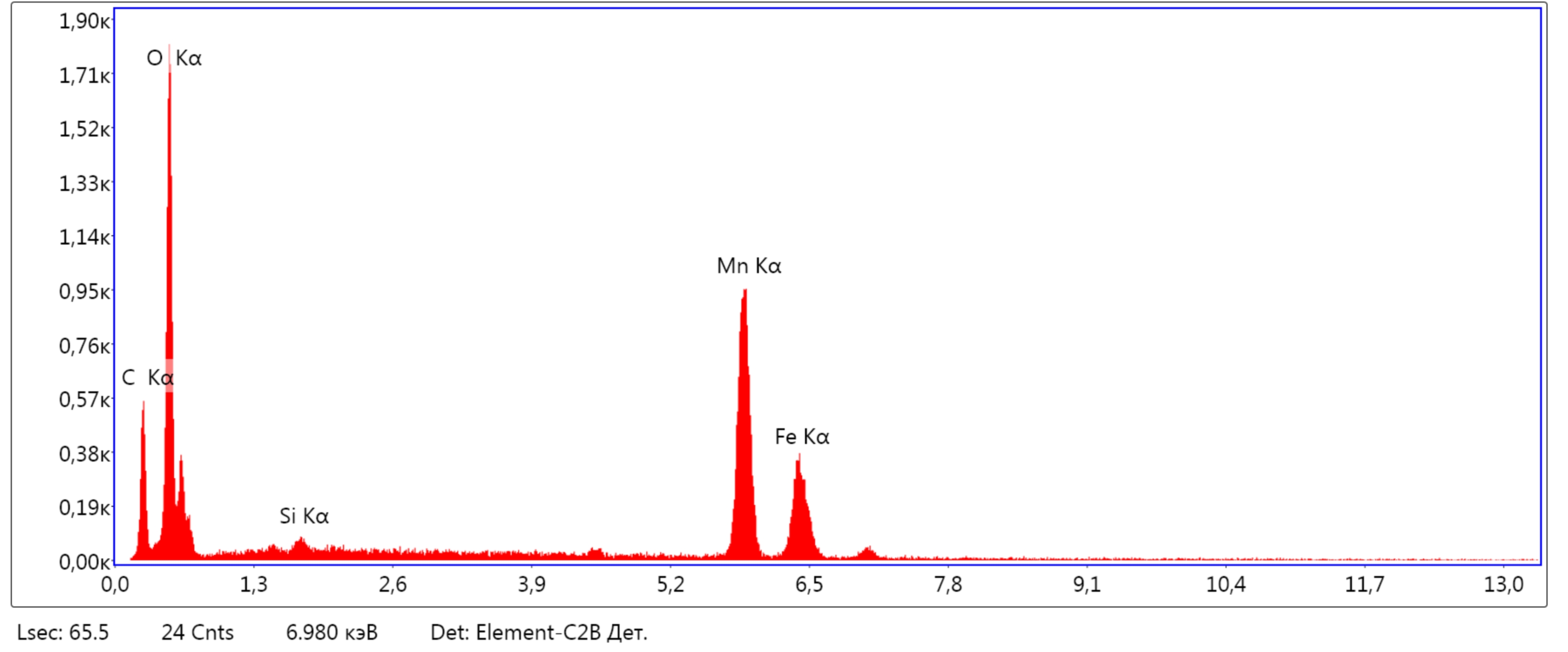


Fig. S2. Results of EDS of a steel disk surface after laser short-pulse processing for a MnO2 – 20% Bi2O3 powder composition


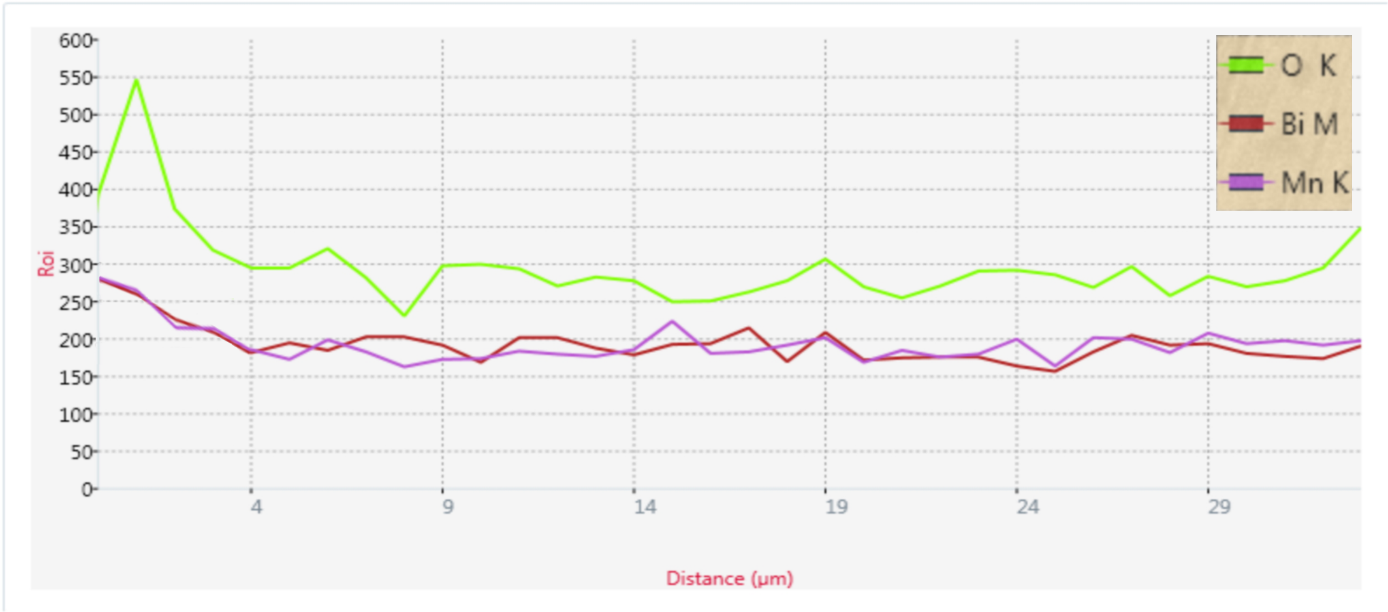


Fig. S3. Results of EDX analysis of the cross-section of a disk after laser treatment and diamond lapping for a MnO_2_ – 20% Bi_2_O_3_ powder composition.


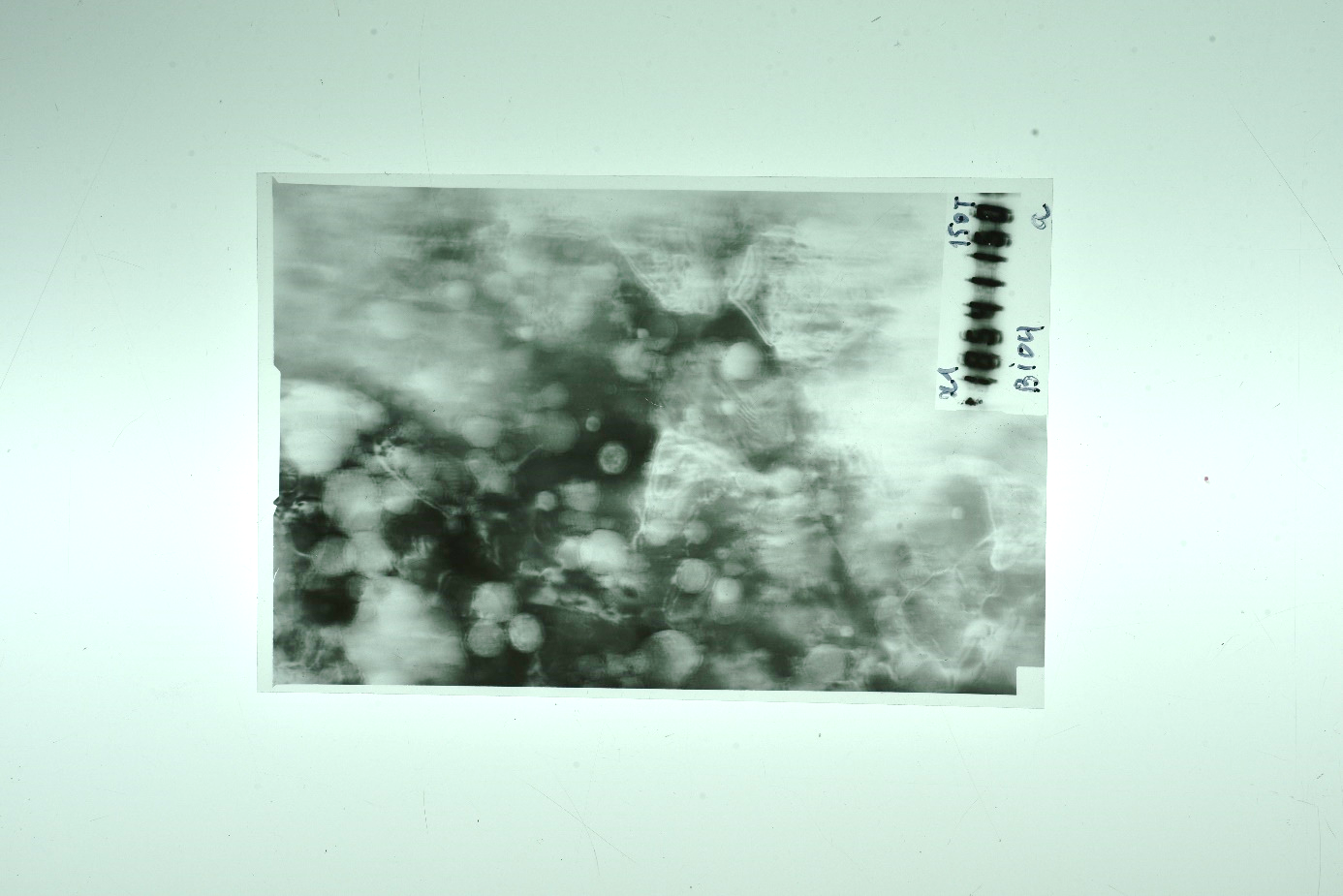

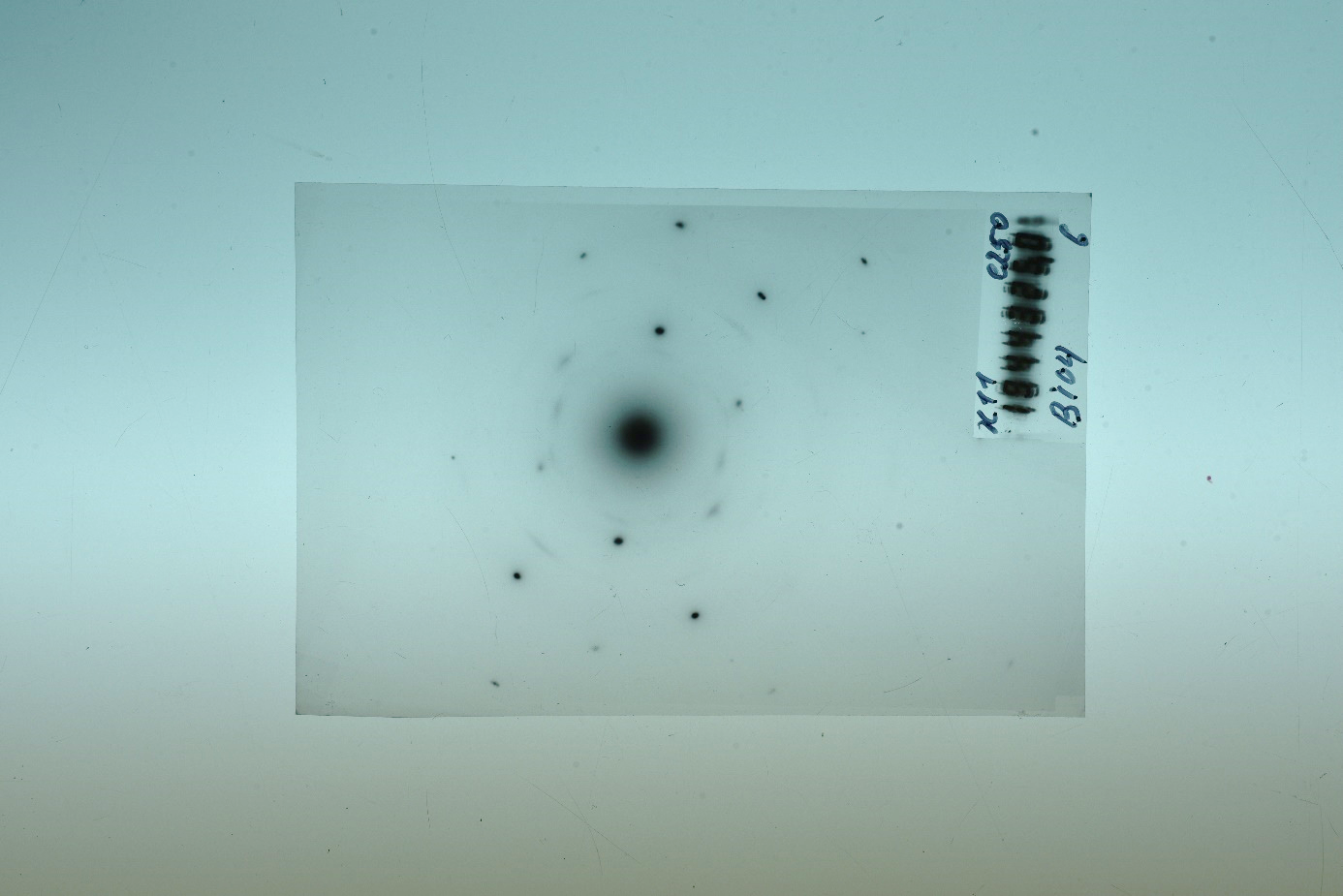


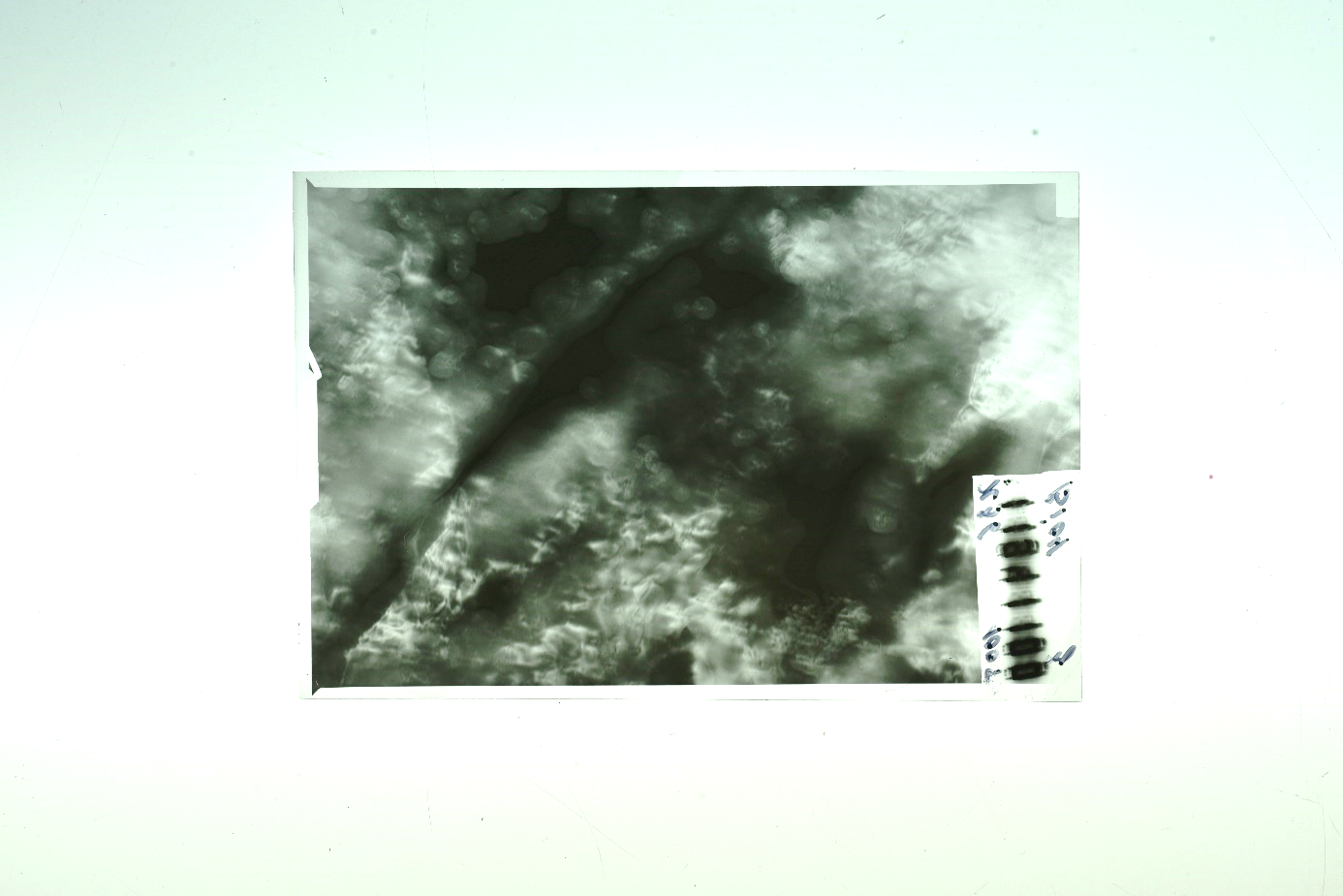

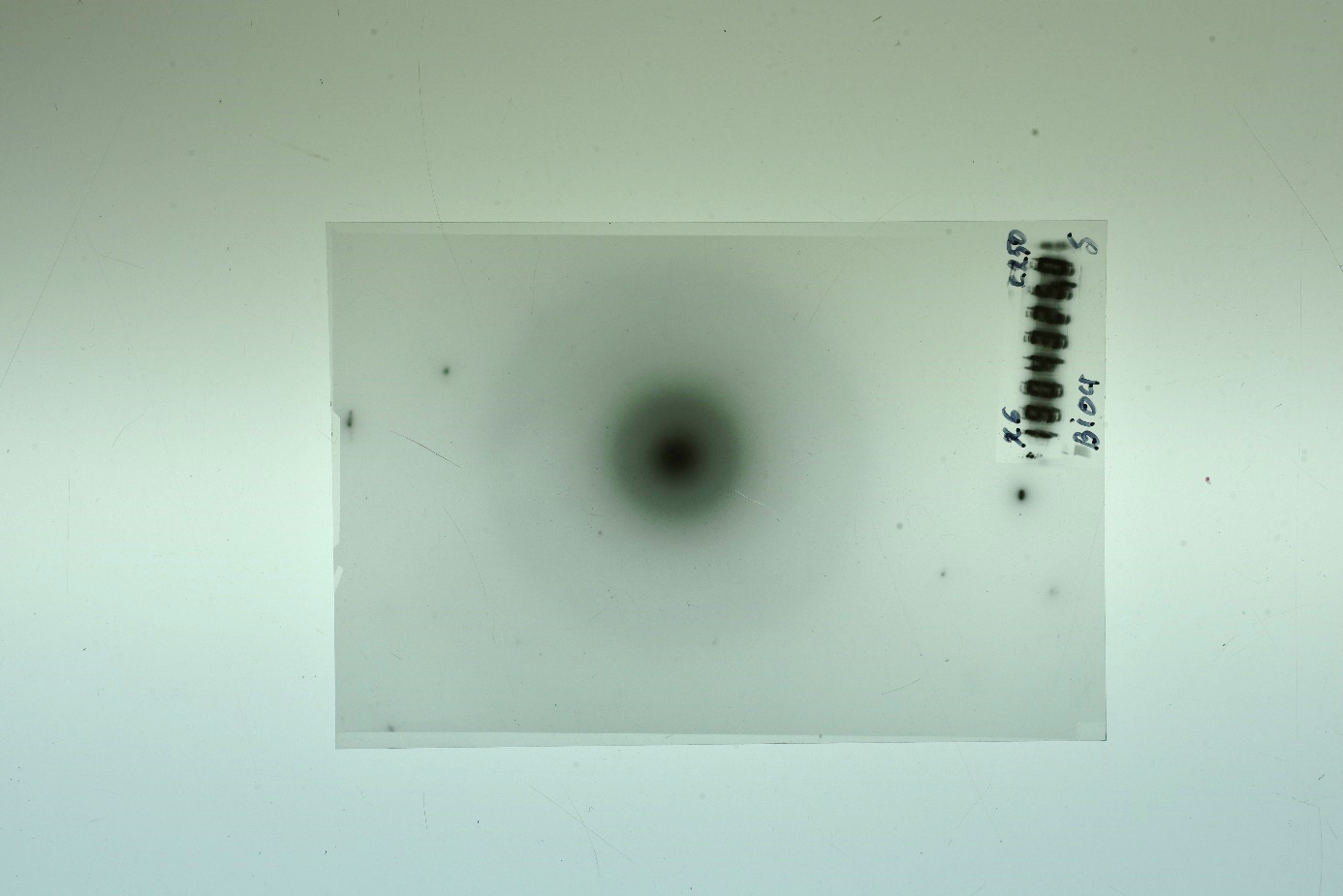


Fig. S4. Film negatives of TEM-images and electron diffraction patterns of different places of a sample subjected to laser processing with a MnO2 – 20% Bi2O3 powder mix


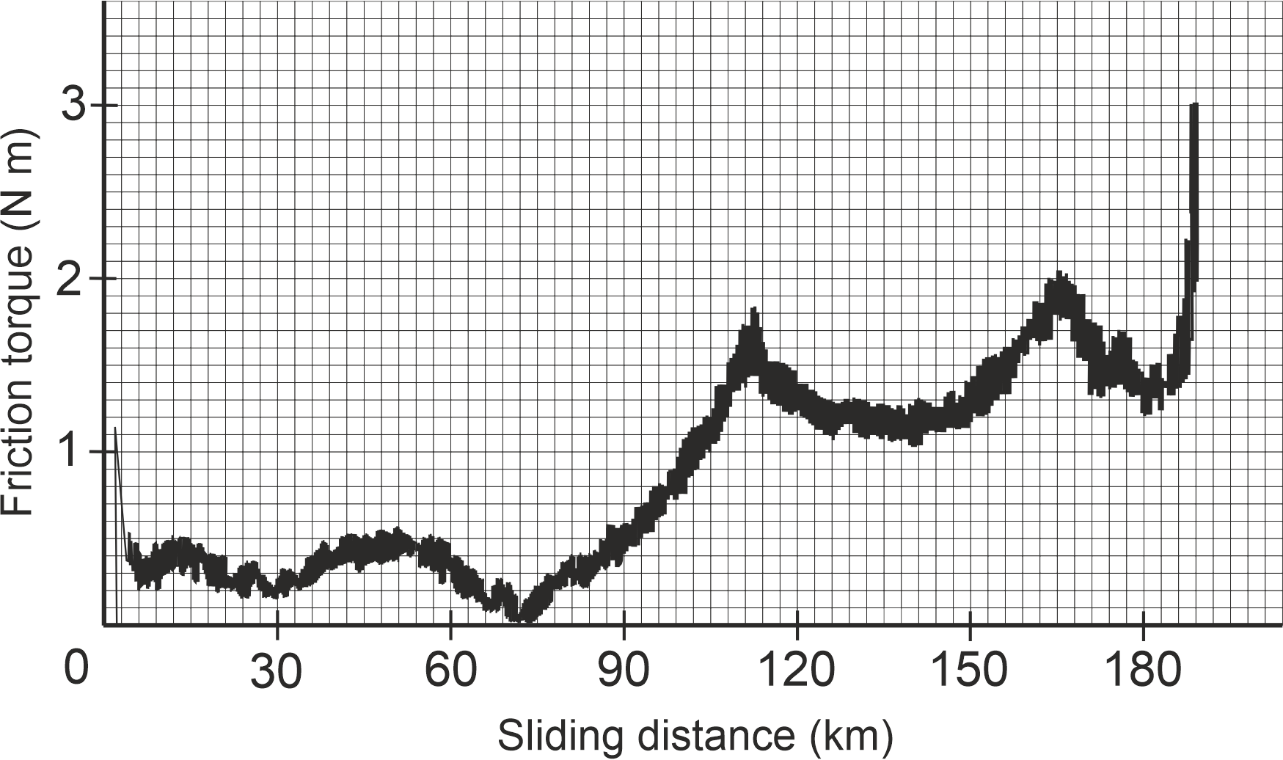


Fig. S5. Raw data of wear test results of MnO_2_ alloyed steel disks sliding against bronze without lubrication at different loads.


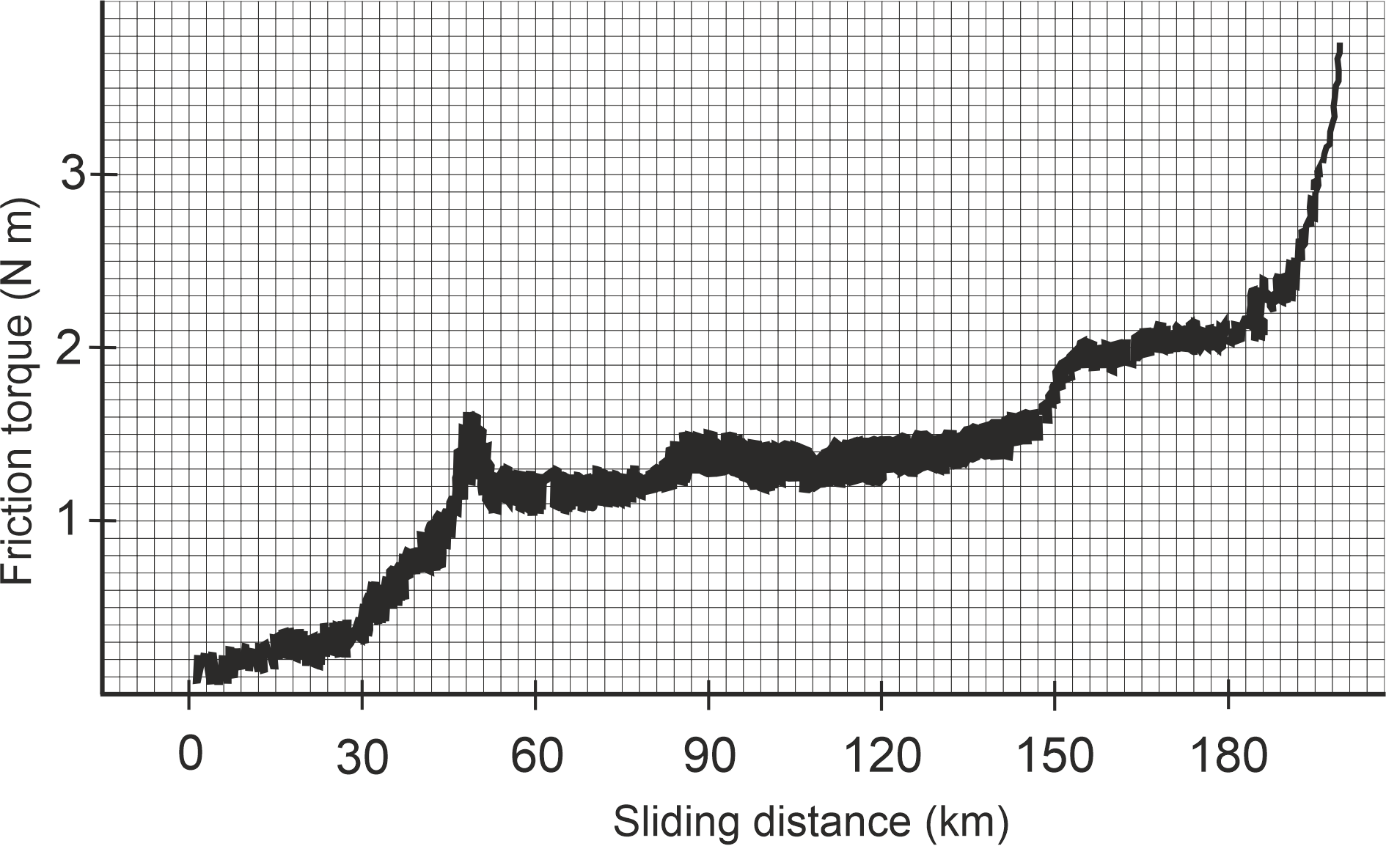


Fig. S6. Raw data of wear test results of MnO_2_ ­– 10%Bi_2_O_3_ alloyed steel disks sliding against bronze without lubrication at different loads.


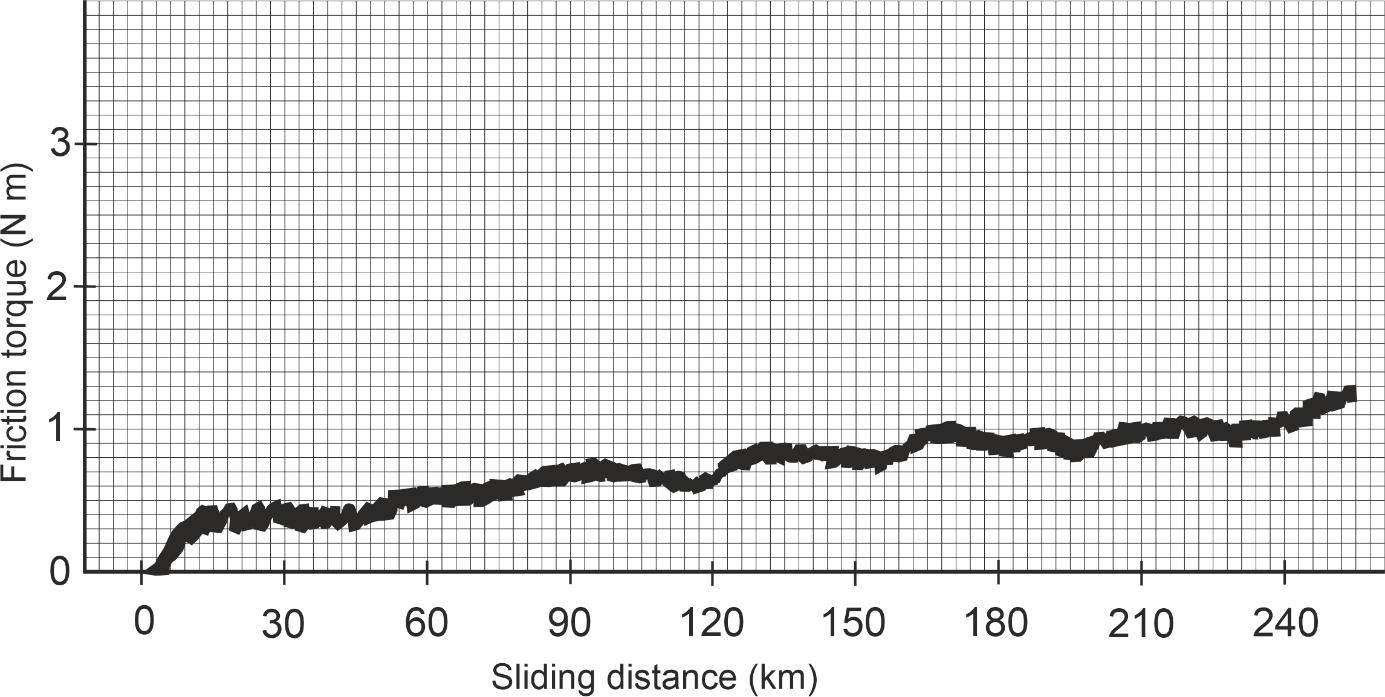


Fig. S7. Raw data of wear test results of MnO_2_ ­– 20%Bi_2_O_3_ alloyed steel disks sliding against bronze without lubrication at different loads.


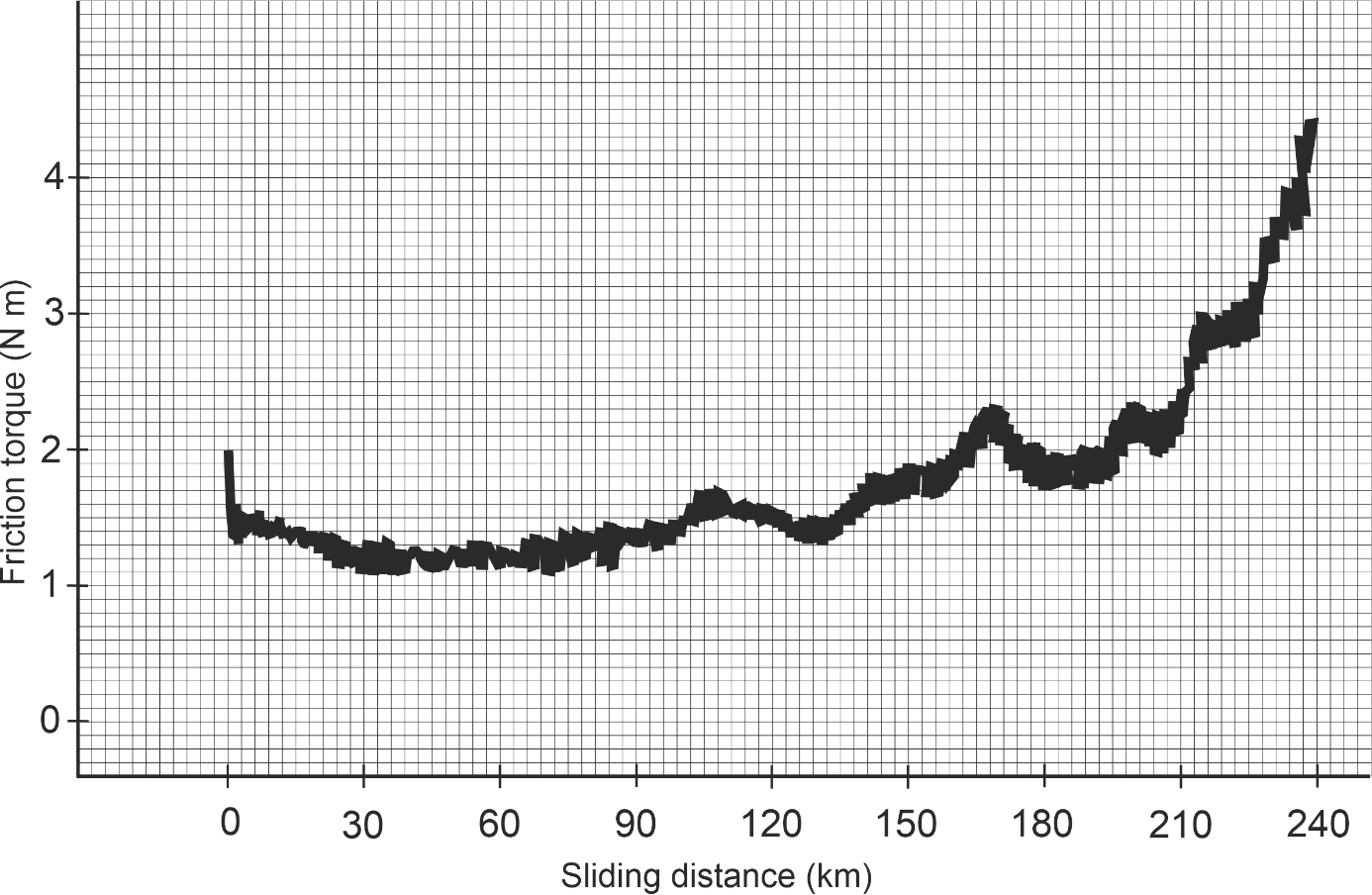


Fig. S8. Raw data of wear test results of MnO_2_ ­– 30%Bi_2_O_3_ alloyed steel disks sliding against bronze without lubrication at different loads: horizontal scale is 0.1 N m per mm; vertical scale is 4.36 m per mm.


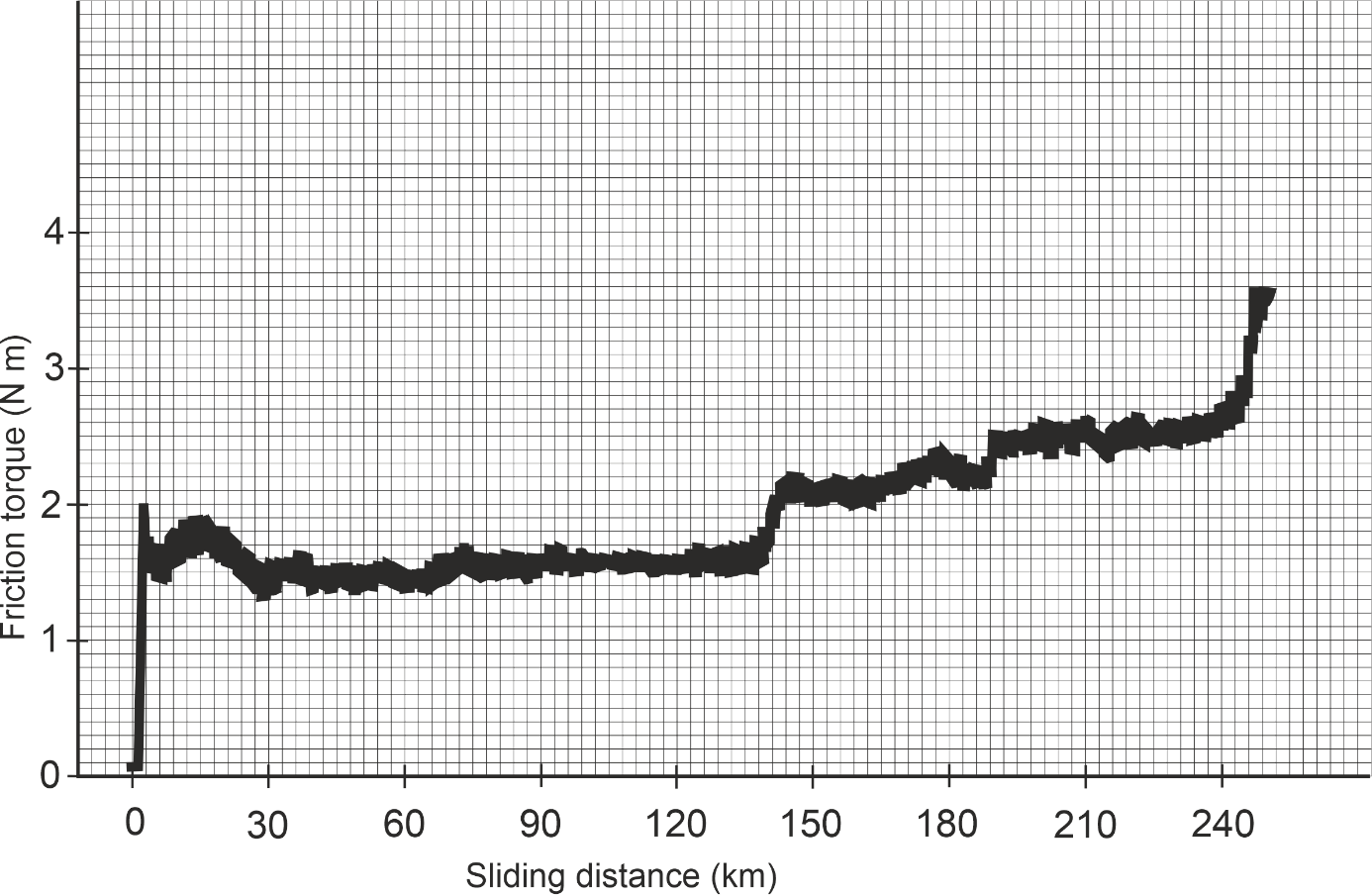


Fig. S9. Raw data of wear test results of MnO_2_ ­– 60%Bi_2_O_3_ alloyed steel disks sliding against bronze without lubrication at different loads: horizontal scale is 0.1 N m per mm; vertical scale is 4.36 m per mm.

**RAW DATA and unproofreaded TEXT of XPS-analyses results**

Two groups of samples were studied by XPS:

1. Pair 1. Steel 40X with Mn - O coating / bronze.

2. Pair 2. 40X steel coated with Mn - O + Bi 2 O 3 / bronze.

**Pair No. 1** (inscription on the package “ MnO ”)

Steel sample from pair (package) 1. The axial region of the convex surface was studied.

In Fig. Figure 1-1 shows the overview spectra of the friction surface of steel from package No. 1.


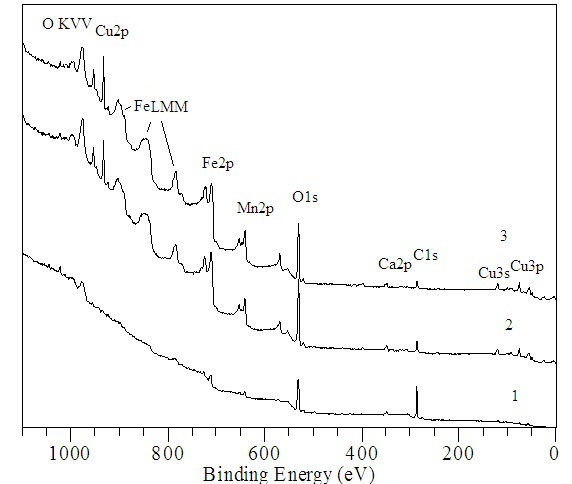


Fig.1-1. Survey spectra of the steel surface: 1 – initial surface, 2 – ion etching 2 min., 3 – ion etching 5 min.

Ca 2 p spin doublet is observed . As etching progresses, the signs of copper clearly increase (peaks of core levels and Auger series).

Element concentrations (at.%), determined from XPS data taking into account photoionization cross sections with background subtraction by the Shirley method , are presented in the table:

| Etching, min. | C | O | Mn | Fe | Zn | N | Ca | Cu |
| --- | --- | --- | --- | --- | --- | --- | --- | --- |
| 0 | 52.6 | 31.6 | 3.1 | 7.4 | 1.1 | 1.1 | 1.4 | 1.6 |
| 2 | 16.6 | 40.5 | 10.3 | 21.6 | 0.9 | 3.0 | 2.0 | 5.1 |
| 5 | 12.6 | 37.9 | 12.0 | 25.3 | 0.4 | 4.9 | 1.3 | 5.6 |

The ratio of element concentrations excluding carbon and oxygen is presented in the table:

| Etching, min . | Mn | Fe | Zn | Ca | Cu |
| --- | --- | --- | --- | --- | --- |
| 0 | 21.4 | 50.4 | 7.8 | 9.2 | 11.2 |
| 2 | 25.9 | 54.2 | 2.2 | 5.0 | 12.7 |
| 5 | 27.0 | 56.7 | 0.8 | 3.0 | 12.5 |

Impurities from the steel composition (for example, silicon) are observed in trace quantities.

Detailed spectra of a steel sample from pair (package) 1:


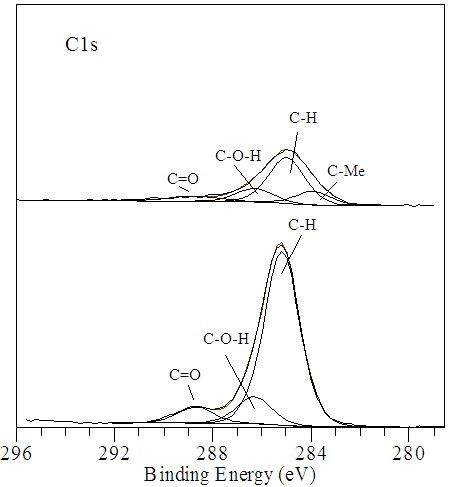


Fig.1-2. C 1 s spectra of the steel surface from pair 1. 1 (lower spectrum) – surface without etching, 2 ( upper spectrum) – surface after etching for 2 min.

In contrast to the spectra obtained earlier on the plate, here we observe the appearance and growth of a peak in the region of 283.5-283.9 eV, characteristic of the formation of C-Me/ bonds.


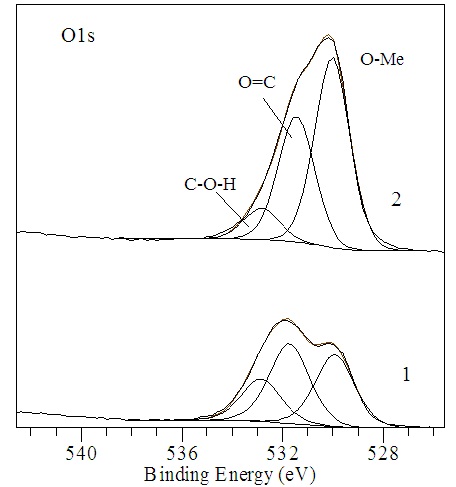


Fig. 1-3. O1 s spectra of steel surface pair 1. Initial surface (1), after etching 2 min (2)

The contribution to the 530 eV region can come from oxygen, which chemically interacts with all detected metal components ( Fe , Mn ). An intense O=C peak is observed in the initial state and after etching for 2 and 5 minutes, in contrast to the spectrum coating on the plate, where after 2 minutes of etching only the Me-O peak remains.


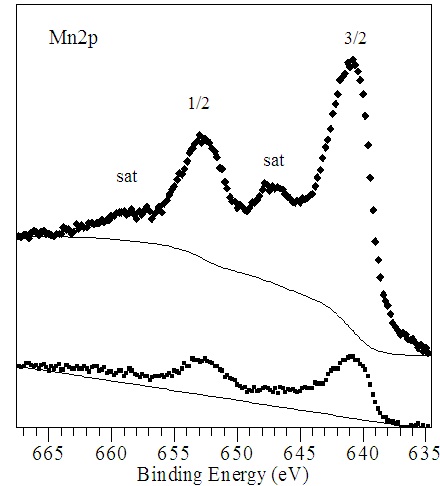


Fig. 1-4. Spectra of manganese Mn 2 p surface in the initial state (1) and after etching for 2 minutes (2).

Intense satellites ~ +6 eV from the main peaks indicate a +2 charge state of manganese.


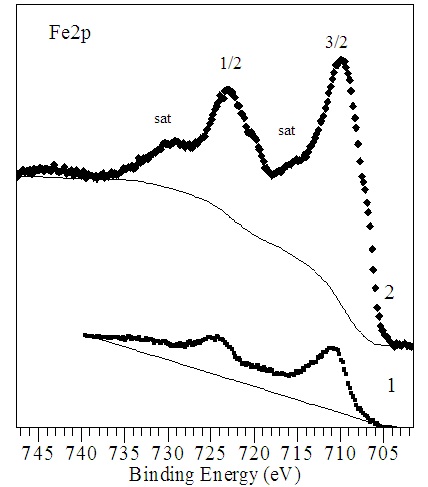


Fig. 1-5. Spectra of Fe 2 p iron surface of steel in the initial state (1) and after etching the surface for 2 minutes (2). Clearly pronounced satellites after 2 min of etching suggest the predominance of Fe 2+ in the coating.


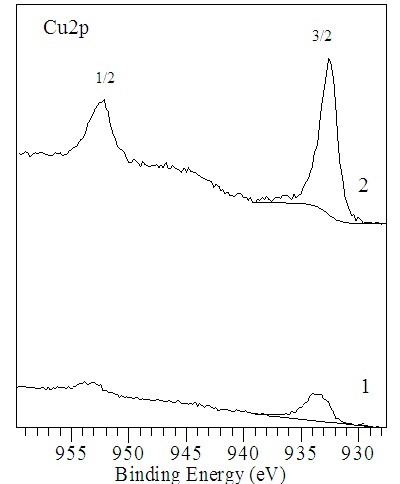


Fig. 1-6. Cu 2 p spectra of the initial steel surface (1) and after ion etching for 2 min (2).

Eb ( Cu 2 p _3/2_ ) of the steel surface before etching has a value of 933.5 eV, that is, copper is oxidized, presumably to _Cu_ 2+ . But at the same time, these spectra do not contain an intense satellite in the region Eb ~ 942 eV, characteristic of Cu 2+ in pure copper monoxide. Further, as etching progresses, this peak ( Cu 2 p 3/2) takes on the value Eb = _932.6_ eV, characteristic of metallic copper, however, the parameters of the Auger peak of Cu LVV differs from the parameters characteristic of unoxidized (pure) copper. It is likely that Cu + is present here .

**Bronze** . The friction surface (wear area) was studied on a bronze sample from package No. 1. The measurements were carried out in MgK α excitation to avoid superposition of the Auger series of copper ( Cu LMM ) to the region of the Fe 2 p and Mn 2 p spectra . In Fig. 1-7 show the overview spectra of a bronze sample without ion etching (1) and after ion etching for 2 minutes (2).


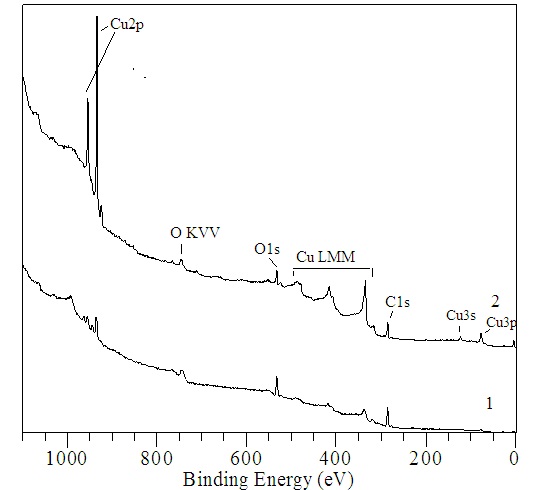


Fig. 1-7. Survey spectra of the friction region of bronze from pair 1.

Initial surface (1), etching 2 min (2)

A detailed analysis of areas characteristic of Fe and Mn showed that “spreading” of the coated steel material on bronze does not occur. From the bronze alloy, it is possible to determine the concentration of aluminum in an amount of ~ 3.3 at.%. Silicon is detected in trace amounts ~ 0.5 at.% and iron (no more than 1.5 at.%). The remaining impurities are not detected at the sensitivity level of the XPS method.

Pair (packet ) No. 2.

**Steel.** Shooting using AlKα anode .


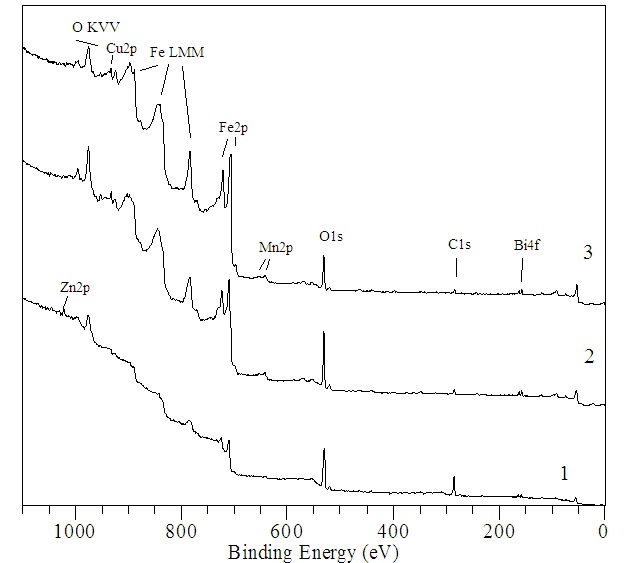


Fig. 2-1. Survey spectra of a steel sample from a pair (package 2): 1 – initial surface, 2 – etching for 2 min., 3 – etching for 5 min.

The concentrations of elements (at.%) in steel from package 2, determined from XPS data taking into account photoionization cross sections and when subtracting the background using the Shirley method , are presented in the table:

| Etching time, min | C | O | Mn | Fe | Zn | Bi | Ca | Cu |  |
| --- | --- | --- | --- | --- | --- | --- | --- | --- | --- |
| 0 | 39.8 | 41.9 | 1.3 | 12.9 | 1.2 | 0.4 | 1.1 | 1.3 |  |
| 2 | 10.1 | 41.4 | 3.3 | 41.7 | 0.5 | 0.5 | 0.8 | 1.7 |  |
| 5 | 9.7 | 28.9 | 3.3 | 54.5 | 0.0 | 0.5 | 0.8 | 2.4 |  |

The ratio of element concentrations excluding carbon and oxygen is presented in the table:

| Etching time, min | Mn | Fe | Zn | Bi | Ca | Cu |
| --- | --- | --- | --- | --- | --- | --- |
| 0 | 7.1 | 70.7 | 6.8 | 2.2 | 6.0 | 7.2 |
| 2 | 6.7 | 86.0 | 1.1 | 1.1 | 1.5 | 3.6 |
| 5 | 5.3 | 88.6 | 0.0 | 0.8 | 1.3 | 4.0 |

As can be seen from the survey spectra and assessment of the quantitative composition of the surface layers, the basis of the coating on steel from package No. 2 consists of iron-based oxides with dissolved manganese cations and a minimal degree of bismuth. Calcium and silicon impurities are found in the composition of the surface layers. As a result of contact with the rubbing mating part, a small amount of copper penetrates into the surface layer of the steel.


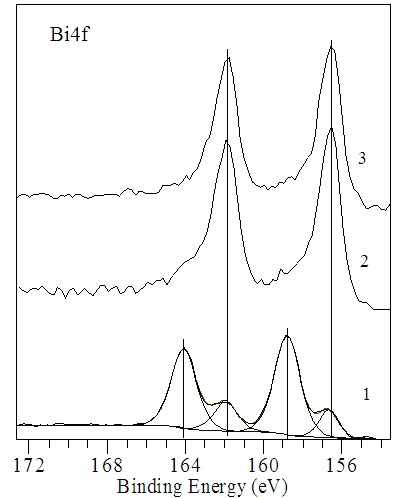


Fig. 2-2. 4 f spectra of bismuth.

1 – initial surface, 2 – etching 2 min, 3 – etching 5 min.

Feature (difference between the spectra of steel from pair 2 and the spectra of the plate ): already on the initial surface before ion etching, along with the 4 f spin doublet from Bi 3+ (158.8 - 164.1 eV), a spin doublet with E st 156 is observed, 6 – 161.9 eV (±0.2 eV), related to photoemission from bismuth in the “metallic” ( Bi ^0^ ) state.


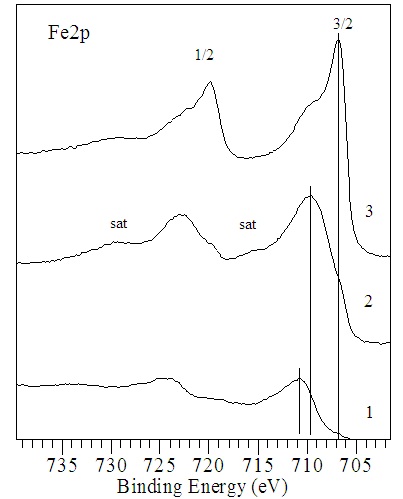


Fig. 2-3. 2p spectra of iron.

1 – initial surface, 2 – etching 2 min, 3 – etching 5 min.

Before ion etching, the spectrum is characteristic of defective spinel based on Fe (3+,2+) _3_ O _4_ of variable composition with the introduction of Mn 2+ and Mn 3+ cations. After etching for 2 min, Fe 2+ predominates .


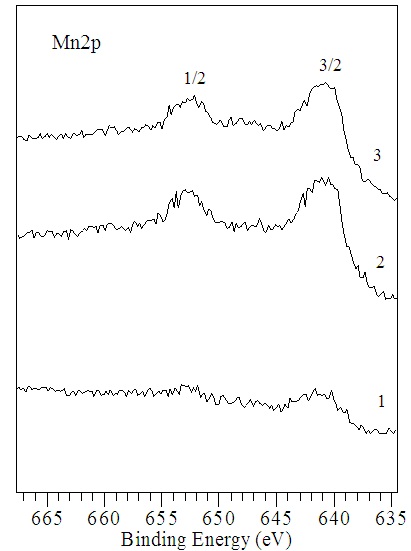


Fig. 2-4. 2p spectra of manganese.

1 – initial surface, 2 – etching 2 min, 3 – etching 5 min.


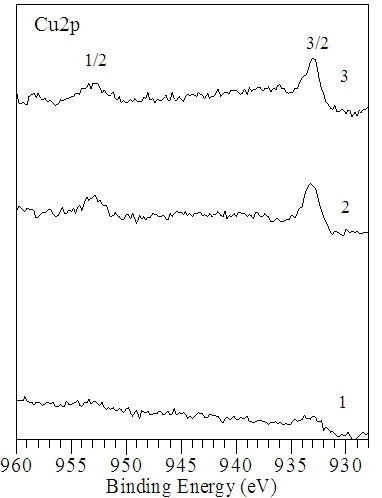


Fig. 2-5. Cu 2p spectra of copper superimposed on the background from the Auger spectrum of Mn LMM . The energy distance between the 2p _3/2_ and 2p _1/2_ peaks is ~19.8 eV.


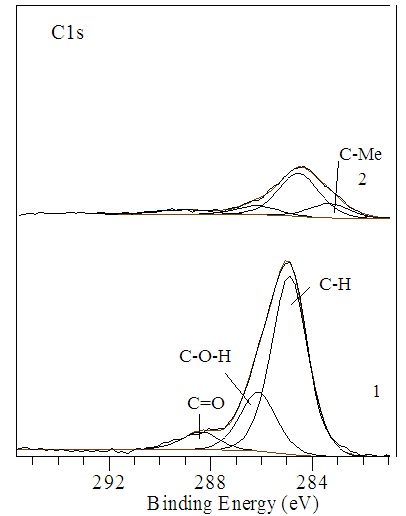


Fig. 2-6. C1 s spectra of steel from package 2.

1 – initial surface, 2 – surface after etching 2 min.

In contrast to the spectra obtained earlier on the plate, here, as on steel from pair 1, the appearance and growth of a peak in the region of 283.5-283.9 eV, characteristic of the formation of C-Me bonds, is observed.


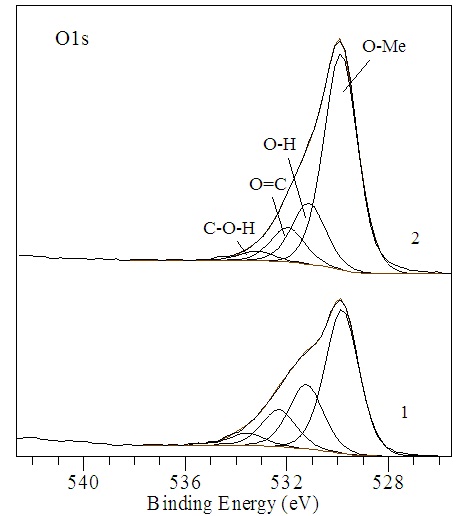


Fig. 2-7. O 1 s spectra of the steel surface from package 2.

1 – surface without ion etching, 2 – surface after etching for 2 min.

The O1 s spectrum before and after etching has a pronounced multicomponent nature. The main intense peak has an Eb value near 530.0 eV and corresponds to oxygen entering directly into the structures of metal oxides. The peak at 531.0 eV should be attributed to oxygen in the O–H groups, the peaks at 532 and 533 eV are attributed to oxygen in the C–O and C – O – H bonds , respectively. In a number of publications, the peak near 533 eV is attributed to structural water, which is always present on the surface of oxides. Note that the oxygen spectrum of pair 2 steel differs from the O 1 s spectrum of pair 1 steel, and has common parameters with the spectrum of a thin plate. But after etching for 2 minutes, only the peak at 530 eV remained on the plate, while here the multicomponent nature of the O 1 s spectrum is preserved.

**Conclusion on the surface of steel from pair (package) 2:**

The surface friction layer consists predominantly of iron oxides 2+ and 3+ of variable composition, into the structure of which manganese cations and (on the surface itself) Bi 3+ cations are embedded. The depth of bismuth oxidation is less than the depth of XPS analysis. During friction, copper ( Cu 1+ impurities) is introduced into the composition of the surface layer. In general, the depth of oxidation of the surface layer of steel pair 2 is insignificant (i.e., comparable to the ultrathin oxide film naturally formed during low-temperature oxidation under normal conditions) and during short-term etching in the spectrum of Fe 2 p , signs of Fe 2+ predominate, and also appear signs of metallic ( Fe ^0^ ) iron.

**Bronze friction surface from pair 2.**

The friction surface (wear area) was studied on a bronze sample from a package (pair)

No. 2. The measurements were carried out in MgK α excitation to avoid superposition of the Auger series of copper ( Cu LMM ) to the region of the Fe 2 p and Mn 2 p spectra .

**
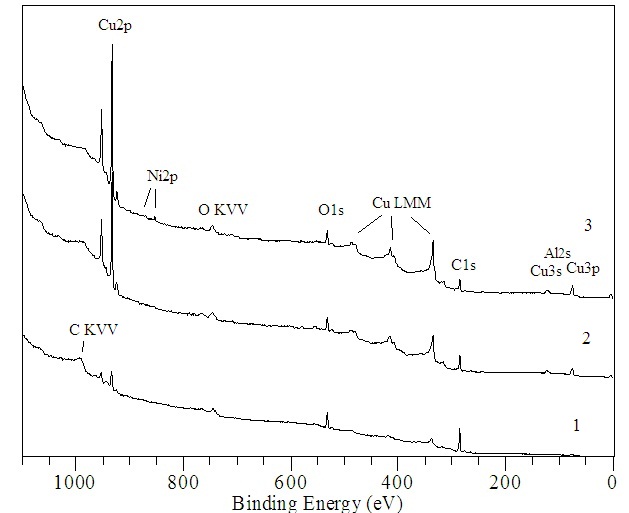
**

Fig. 2-6. Survey spectra of the bronze friction section from package 2.

1. initial surface, 2 – etching 2 min, 3 – etching 5 min.

The elemental composition of the friction surface on bronze was assessed (see table).

Table. Elemental composition (at.%) of the friction area on bronze from package No. 2.

| Etching time, min | Al | Si | Fe | C | O | Cu | Bi | Mn | Ni |
| --- | --- | --- | --- | --- | --- | --- | --- | --- | --- |
| 0 | 0.0 | 0.0 | 0.0 | 65.9 | 21.1 | 13.0 | 0 | - |  |
| 2 | 5.0 | 3.9 | 1.0 | 40.2 | 16.0 | 34.0 | 0 | - |  |
| 5 | 6.1 | 0.0 | 1.3 | 32.5 | 17.0 | 43.1 | 0 | - | sl |

A detailed analysis of areas characteristic of Fe , Mn and Bi showed that “spreading” of the coated steel material onto bronze does not occur during friction.

**MODEL AND TYPE OF TESTED TURBOCHARGERS**


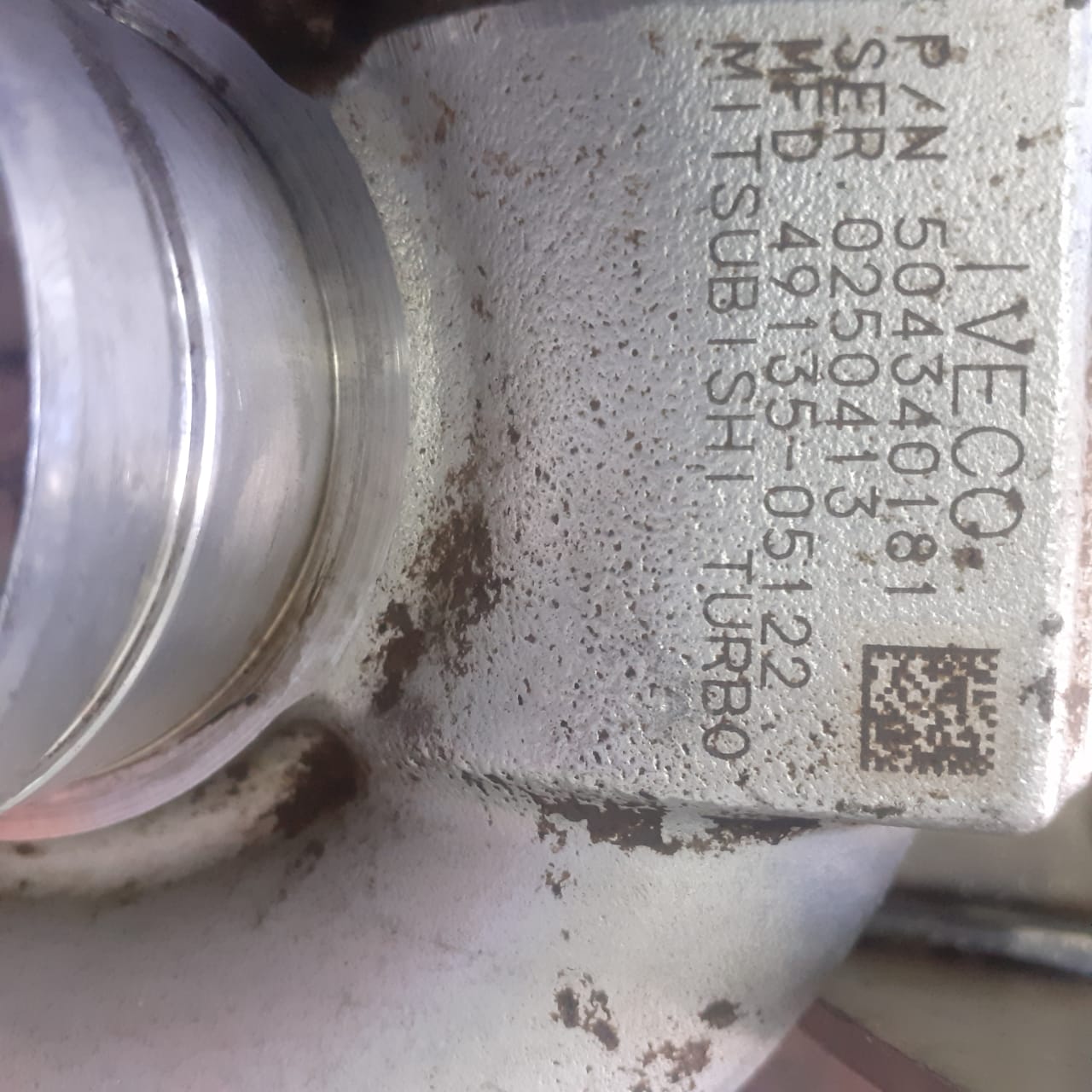


Fig. S10. Tested turbochargers


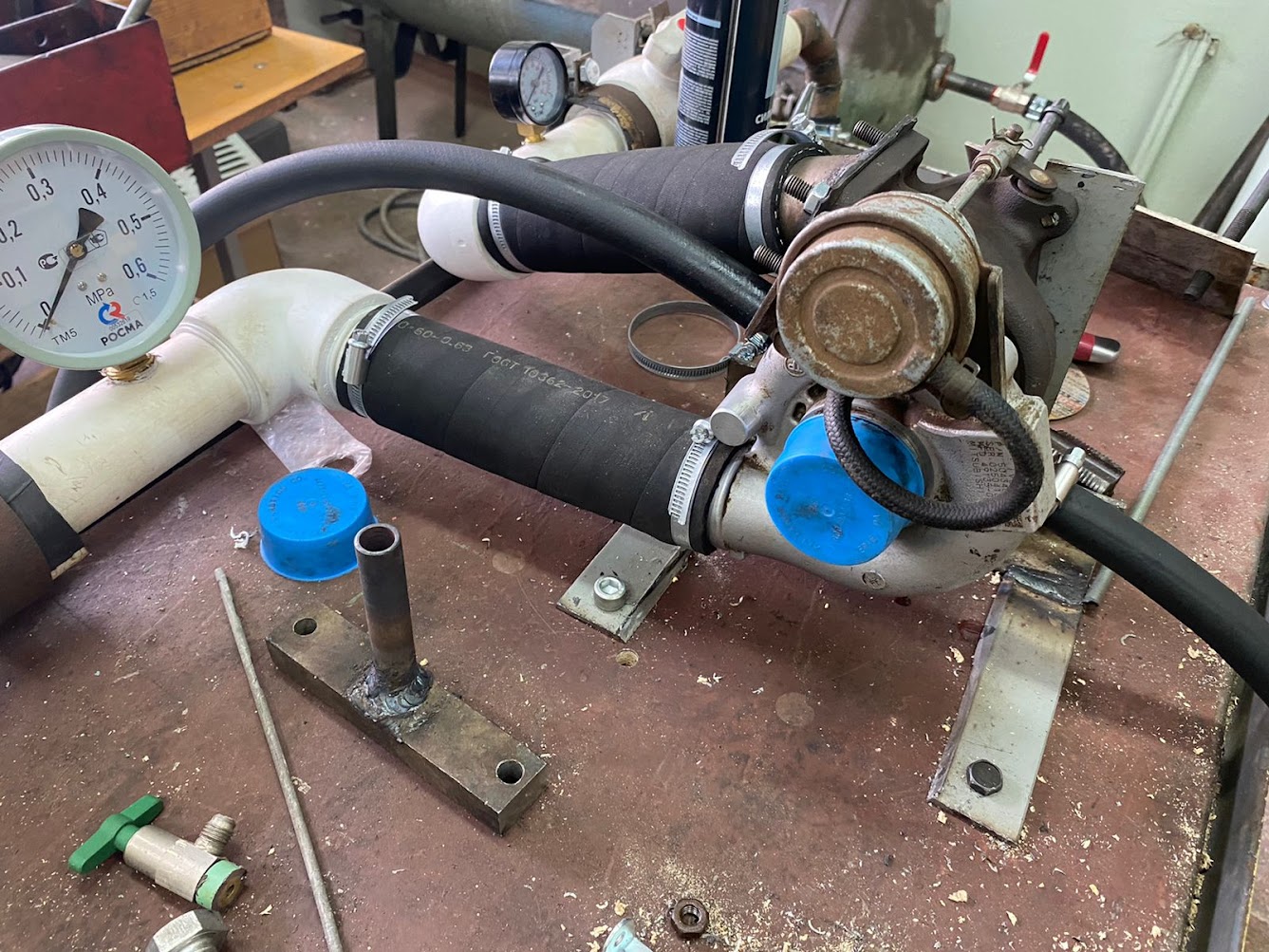


Fig. S11. Turbochargers test platform. Side view


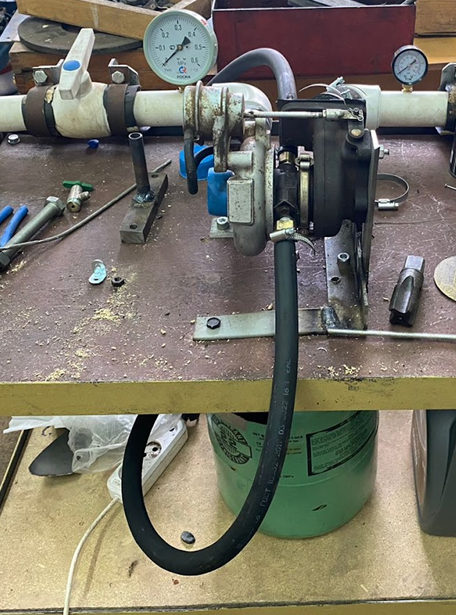


Fig. S12. Turbochargers test platform. Front view

RAW DATA for Rotation stoppage time measurements with unmodified and modified turbochargers

Time-measurement accuracy: ± 0.5 s

| Test N | Rotation stoppage time of unmodified turbocharger, s | Rotation stoppage time of modified turbocharger, s |
| --- | --- | --- |
|  |  |  |
| 1 | 5.1 | 7.2 |
| 2 | 4.2 | 8.1 |
| 3 | 4 | 9.3 |
| 4 | 3.3 | 10.4 |
| 5 | 2.9 | 11 |
| 6 |  | 11.5 |
| 7 |  | 11.8 |
| 8 |  | 11 |
| 9 |  | 11.6 |
| 10 |  | 11.8 |
| 11 |  | 11.9 |
| 12 |  | 11 |
| 13 |  | 11.6 |
| 14 |  | 11.7 |
| 15 |  | 12 |
| 16 |  | 12 |
| 17 |  | 11.1 |
| 18 |  | 11.4 |
| 19 |  | 11.2 |
| 20 |  | 12 |
| 21 |  | 11.4 |
| 22 |  | 11.9 |
| 23 |  | 11.2 |
| 24 |  | 11.9 |
| 25 |  | 11.6 |
| 26 |  | 11.3 |
| 27 |  | 11.1 |
| 28 |  | 11.2 |
| 29 |  | 11.1 |
| 30 |  | 11.2 |
| 31 |  | 11.3 |
| 32 |  | 11 |
| 33 |  | 11.7 |
| 34 |  | 11 |
| 35 |  | 11.8 |
| 36 |  | 11.6 |
| 37 |  | 11.7 |
| 38 |  | 11.1 |
| 39 |  | 11.8 |
| 40 |  | 11.9 |
| 41 |  | 11 |
| 42 |  | 11.3 |
| 43 |  | 11.6 |
| 44 |  | 11.6 |
| 45 |  | 11 |
| 46 |  | 11.6 |
| 47 |  | 11.1 |
| 48 |  | 11.3 |
| 49 |  | 11.5 |
| 50 |  | 11.7 |
| 51 |  | 12 |
| 52 |  | 11 |
| 53 |  | 11.1 |
| 54 |  | 12 |
| 55 |  | 11.4 |
| 56 |  | 11.2 |
| 57 |  | 11.1 |
| 58 |  | 11.3 |
| 59 |  | 11.8 |
| 60 |  | 11 |
| 61 |  | 11.9 |
| 62 |  | 12 |
| 63 |  | 11.6 |
| 64 |  | 11.7 |
| 65 |  | 11.7 |
| 66 |  | 11.7 |
| 67 |  | 11.1 |
| 68 |  | 12 |
| 69 |  | 11.6 |
| 70 |  | 11.2 |
| 71 |  | 11.2 |
| 72 |  | 11.2 |
| 73 |  | 12 |
| 74 |  | 11.4 |
| 75 |  | 11.6 |
| 76 |  | 11.5 |
| 77 |  | 11.7 |
| 78 |  | 11.3 |
| 79 |  | 11.8 |
| 80 |  | 11 |
| 81 |  | 11.1 |
| 82 |  | 11.3 |
| 83 |  | 11.9 |
| 84 |  | 11.7 |
| 85 |  | 11 |
| 86 |  | 12 |
| 87 |  | 11.3 |
| 88 |  | 11.5 |
| 89 |  | 11.5 |
| 90 |  | 11.5 |
| 91 |  | 11.8 |
| 92 |  | 11.1 |
| 93 |  | 11.6 |
| 94 |  | 11.4 |
| 95 |  | 11.2 |
| 96 |  | 11.7 |
| 97 |  | 11.4 |
| 98 |  | 11 |
| 99 |  | 11 |
| 100 |  | 11.7 |
| 101 |  | 11.9 |
| 102 |  | 11.8 |
| 103 |  | 11.7 |
| 104 |  | 12 |
| 105 |  | 11.3 |
| 106 |  | 11 |
| 107 |  | 11.6 |
| 108 |  | 11.6 |
| 109 |  | 11.8 |
| 110 |  | 11.5 |
| 111 |  | 11.3 |
| 112 |  | 11.2 |
| 113 |  | 11.5 |
| 114 |  | 11.2 |
| 115 |  | 11.1 |
| 116 |  | 11.5 |
| 117 |  | 11 |
| 118 |  | 11.7 |
| 119 |  | 11.7 |
| 120 |  | 11.4 |
| 121 |  | 11.3 |
| 122 |  | 11.4 |
| 123 |  | 11.8 |
| 124 |  | 12 |
| 125 |  | 11.8 |
| 126 |  | 11.3 |
| 127 |  | 11.6 |
| 128 |  | 11.2 |
| 129 |  | 11 |
| 130 |  | 11.4 |
| 131 |  | 11.1 |
| 132 |  | 12 |
| 133 |  | 11.7 |
| 134 |  | 11.6 |
| 135 |  | 11.1 |
| 136 |  | 11.6 |
| 137 |  | 11.4 |
| 138 |  | 11.5 |
| 139 |  | 11.3 |
| 140 |  | 11.3 |
| 141 |  | 11.7 |
| 142 |  | 11.9 |
| 143 |  | 11.8 |
| 144 |  | 12 |
| 145 |  | 11.8 |
| 146 |  | 11.6 |
| 147 |  | 11.4 |
| 148 |  | 11.7 |
| 149 |  | 11.3 |
| 150 |  | 11 |
| 151 |  | 11.9 |
| 152 |  | 12 |
| 153 |  | 12 |
| 154 |  | 11.8 |
| 155 |  | 11.5 |
| 156 |  | 11.4 |
| 157 |  | 11.3 |
| 158 |  | 12 |
| 159 |  | 11.7 |
| 160 |  | 11.2 |
| 161 |  | 11 |
| 162 |  | 11.3 |
| 163 |  | 11.3 |
| 164 |  | 11.6 |
| 165 |  | 11.5 |
| 166 |  | 11.3 |
| 167 |  | 11.1 |
| 168 |  | 11.9 |
| 169 |  | 11.7 |
| 170 |  | 11.1 |
| 171 |  | 11.6 |
| 172 |  | 11 |
| 173 |  | 11.8 |
| 174 |  | 11.8 |
| 175 |  | 11.4 |
| 176 |  | 11 |
| 177 |  | 11.2 |
| 178 |  | 11.5 |
| 179 |  | 11.6 |
| 180 |  | 11.3 |
| 181 |  | 11 |
| 182 |  | 11.8 |
| 183 |  | 11.5 |
| 184 |  | 11 |
| 185 |  | 11.8 |
| 186 |  | 11.9 |
| 187 |  | 11.6 |
| 188 |  | 11.8 |
| 189 |  | 11.9 |
| 190 |  | 11.2 |
| 191 |  | 11.9 |
| 192 |  | 11.5 |
| 193 |  | 11.7 |
| 194 |  | 12 |
| 195 |  | 11.5 |
| 196 |  | 11.6 |
| 197 |  | 11.2 |
| 198 |  | 11 |
| 199 |  | 11.2 |
| 200 |  | 11 |
| 201 |  | 10.6 |
| 202 |  | 10.9 |
| 203 |  | 10.6 |
| 204 |  | 10.5 |
| 205 |  | 10.5 |
| 206 |  | 10.8 |
| 207 |  | 10.4 |
| 208 |  | 10.6 |
| 209 |  | 10.3 |
| 210 |  | 10.8 |
| 211 |  | 10.2 |
| 212 |  | 10.4 |
| 213 |  | 10.2 |
| 214 |  | 10.6 |
| 215 |  | 10.2 |
| 216 |  | 10.7 |
| 217 |  | 10.7 |
| 218 |  | 10.7 |
| 219 |  | 10.4 |
| 220 |  | 10.5 |
| 221 |  | 10.3 |
| 222 |  | 10.4 |
| 223 |  | 10.6 |
| 224 |  | 10.3 |
| 225 |  | 10.1 |
| 226 |  | 10.5 |
| 227 |  | 10.2 |
| 228 |  | 10.6 |
| 229 |  | 10.3 |
| 230 |  | 10.6 |
| 231 |  | 10 |
| 232 |  | 10.3 |
| 233 |  | 10.7 |
| 234 |  | 10 |
| 235 |  | 10.5 |
| 236 |  | 9.9 |
| 237 |  | 10.5 |
| 238 |  | 10 |
| 239 |  | 10 |
| 240 |  | 10.6 |
| 241 |  | 10.4 |
| 242 |  | 10.6 |
| 243 |  | 10 |
| 244 |  | 9.9 |
| 245 |  | 9.9 |
| 246 |  | 10.2 |
| 247 |  | 9.9 |
| 248 |  | 10 |
| 249 |  | 10.5 |
| 250 |  | 10 |
| 251 |  | 10.2 |
| 252 |  | 10.1 |
| 253 |  | 10.5 |
| 254 |  | 10.4 |
| 255 |  | 10 |
| 256 |  | 10.1 |
| 257 |  | 10 |
| 258 |  | 9.7 |
| 259 |  | 9.9 |
| 260 |  | 10.3 |
| 261 |  | 9.8 |
| 262 |  | 9.7 |
| 263 |  | 9.7 |
| 264 |  | 9.9 |
| 265 |  | 10.2 |
| 266 |  | 10 |
| 267 |  | 9.6 |
| 268 |  | 9.7 |
| 269 |  | 9.6 |
| 270 |  | 9.6 |
| 271 |  | 9.9 |
| 272 |  | 10 |
| 273 |  | 10.2 |
| 274 |  | 9.6 |
| 275 |  | 9.9 |
| 276 |  | 9.8 |
| 277 |  | 9.9 |
| 278 |  | 9.9 |
| 279 |  | 10.2 |
| 280 |  | 10.2 |
| 281 |  | 9.7 |
| 282 |  | 9.7 |
| 283 |  | 10.2 |
| 284 |  | 9.9 |
| 285 |  | 9.5 |
| 286 |  | 10 |
| 287 |  | 9.7 |
| 288 |  | 9.4 |
| 289 |  | 9.7 |
| 290 |  | 9.7 |
| 291 |  | 9.9 |
| 292 |  | 9.5 |
| 293 |  | 10 |
| 294 |  | 9.4 |
| 295 |  | 9.6 |
| 296 |  | 10 |
| 297 |  | 9.8 |
| 298 |  | 9.7 |
| 299 |  | 9.6 |
| 300 |  | 10 |
| 301 |  | 9.4 |
| 302 |  | 9.2 |
| 303 |  | 9.1 |
| 304 |  | 9.9 |
| 305 |  | 9.8 |
| 306 |  | 9.8 |
| 307 |  | 9.8 |
| 308 |  | 9.1 |
| 309 |  | 9.1 |
| 310 |  | 9.7 |
| 311 |  | 9.4 |
| 312 |  | 9.2 |
| 313 |  | 8.9 |
| 314 |  | 9.5 |
| 315 |  | 9.6 |
| 316 |  | 9.4 |
| 317 |  | 9.2 |
| 318 |  | 9 |
| 319 |  | 9.4 |
| 320 |  | 9.1 |
| 321 |  | 9.6 |
| 322 |  | 8.7 |
| 323 |  | 9.5 |
| 324 |  | 8.8 |
| 325 |  | 9.5 |
| 326 |  | 9.5 |
| 327 |  | 9.4 |
| 328 |  | 8.6 |
| 329 |  | 8.7 |
| 330 |  | 9.5 |
| 331 |  | 8.7 |
| 332 |  | 8.7 |
| 333 |  | 9.4 |
| 334 |  | 9.1 |
| 335 |  | 8.8 |
| 336 |  | 9.2 |
| 337 |  | 8.9 |
| 338 |  | 8.9 |
| 339 |  | 9 |
| 340 |  | 8.9 |
| 341 |  | 9.1 |
| 342 |  | 9 |
| 343 |  | 8.5 |
| 344 |  | 8.9 |
| 345 |  | 8.8 |
| 346 |  | 8.9 |
| 347 |  | 9 |
| 348 |  | 9.2 |
| 349 |  | 8.6 |
| 350 |  | 8.6 |
| 351 |  | 9 |
| 352 |  | 9.2 |
| 353 |  | 9 |
| 354 |  | 9.1 |
| 355 |  | 8.3 |
| 356 |  | 9 |
| 357 |  | 8.2 |
| 358 |  | 9.1 |
| 359 |  | 8.8 |
| 360 |  | 8.8 |
| 361 |  | 8.8 |
| 362 |  | 8.2 |
| 363 |  | 8.5 |
| 364 |  | 8.6 |
| 365 |  | 8.4 |
| 366 |  | 9 |
| 367 |  | 8 |
| 368 |  | 8.1 |
| 369 |  | 8.3 |
| 370 |  | 8.5 |
| 371 |  | 8.6 |
| 372 |  | 8.3 |
| 373 |  | 8.3 |
| 374 |  | 8.3 |
| 375 |  | 8.8 |
| 376 |  | 8.7 |
| 377 |  | 7.9 |
| 378 |  | 8.6 |
| 379 |  | 8.1 |
| 380 |  | 8.4 |
| 381 |  | 7.9 |
| 382 |  | 8.5 |
| 383 |  | 8.7 |
| 384 |  | 8.7 |
| 385 |  | 8.4 |
| 386 |  | 8.5 |
| 387 |  | 8.5 |
| 388 |  | 8.5 |
| 389 |  | 8.7 |
| 390 |  | 8.2 |
| 391 |  | 8 |
| 392 |  | 7.6 |
| 393 |  | 8.4 |
| 394 |  | 8.1 |
| 395 |  | 7.8 |
| 396 |  | 8.1 |
| 397 |  | 7.6 |
| 398 |  | 8.3 |
| 399 |  | 7.6 |
| 400 |  | 7.8 |
| 401 |  | 8.1 |
| 402 |  | 7.5 |
| 403 |  | 8.4 |
| 404 |  | 8.3 |
| 405 |  | 7.7 |
| 406 |  | 8.2 |
| 407 |  | 8.4 |
| 408 |  | 7.9 |
| 409 |  | 8 |
| 410 |  | 8.1 |
| 411 |  | 8.1 |
| 412 |  | 7.6 |
| 413 |  | 7.3 |
| 414 |  | 7.6 |
| 415 |  | 7.5 |
| 416 |  | 8 |
| 417 |  | 7.6 |
| 418 |  | 8.1 |
| 419 |  | 8 |
| 420 |  | 7.8 |
| 421 |  | 7.6 |
| 422 |  | 7.4 |
| 423 |  | 8.2 |
| 424 |  | 7.7 |
| 425 |  | 7.1 |
| 426 |  | 7.8 |
| 427 |  | 7.9 |
| 428 |  | 7.5 |
| 429 |  | 7.2 |
| 430 |  | 7.1 |
| 431 |  | 7.5 |
| 432 |  | 7.3 |
| 433 |  | 7.8 |
| 434 |  | 7.3 |
| 435 |  | 7.7 |
| 436 |  | 7.4 |
| 437 |  | 7.8 |
| 438 |  | 7 |
| 439 |  | 7.7 |
| 440 |  | 7.7 |
| 441 |  | 7.1 |
| 442 |  | 7 |
| 443 |  | 7.1 |
| 444 |  | 7.1 |
| 445 |  | 7.2 |
| 446 |  | 6.9 |
| 447 |  | 6.9 |
| 448 |  | 7.5 |
| 449 |  | 6.8 |
| 450 |  | 7.7 |
| 451 |  | 7 |
| 452 |  | 7.2 |
| 453 |  | 7.6 |
| 454 |  | 6.8 |
| 455 |  | 7.6 |
| 456 |  | 7.4 |
| 457 |  | 6.9 |
| 458 |  | 6.6 |
| 459 |  | 6.6 |
| 460 |  | 7.4 |
| 461 |  | 6.7 |
| 462 |  | 7.5 |
| 463 |  | 7.5 |
| 464 |  | 6.5 |
| 465 |  | 6.9 |
| 466 |  | 6.6 |
| 467 |  | 6.7 |
| 468 |  | 6.7 |
| 469 |  | 6.9 |
| 470 |  | 7.5 |
| 471 |  | 6.8 |
| 472 |  | 6.5 |
| 473 |  | 6.4 |
| 474 |  | 6.4 |
| 475 |  | 6.6 |
| 476 |  | 6.7 |
| 477 |  | 6.7 |
| 478 |  | 6.5 |
| 479 |  | 7.2 |
| 480 |  | 7.1 |
| 481 |  | 7.3 |
| 482 |  | 7 |
| 483 |  | 7 |
| 484 |  | 6.5 |
| 485 |  | 7 |
| 486 |  | 6.4 |
| 487 |  | 6.5 |
| 488 |  | 7.1 |
| 489 |  | 6.7 |
| 490 |  | 6.3 |
| 491 |  | 6.5 |
| 492 |  | 6.8 |
| 493 |  | 6.8 |
| 494 |  | 7.1 |
| 495 |  | 6.8 |
| 496 |  | 6.5 |
| 497 |  | 6.9 |
| 498 |  | 6.1 |
| 499 |  | 7 |
| 500 |  | 7 |
